# Adipose Tissue in Persons With HIV Is Enriched for CD4^+^ T Effector Memory and T Effector Memory RA^+^ Cells, Which Show Higher CD69 Expression and CD57, CX3CR1, GPR56 Co-expression With Increasing Glucose Intolerance

**DOI:** 10.3389/fimmu.2019.00408

**Published:** 2019-03-19

**Authors:** Celestine N. Wanjalla, Wyatt J. McDonnell, Louise Barnett, Joshua D. Simmons, Briana D. Furch, Morgan C. Lima, Beverly O. Woodward, Run Fan, Ye Fei, Paxton G. Baker, Ramesh Ram, Mark A. Pilkinton, Mona Mashayekhi, Nancy J. Brown, Simon A. Mallal, Spyros A. Kalams, John R. Koethe

**Affiliations:** ^1^Division of Infectious Diseases, Department of Medicine, Vanderbilt University Medical Center, Nashville, TN, United States; ^2^Center for Translational Immunology and Infectious Disease, Vanderbilt University Medical Center, Nashville, TN, United States; ^3^Vanderbilt Vaccine Center, Vanderbilt University Medical Center, Nashville, TN, United States; ^4^Department of Pathology, Microbiology, and Immunology, Vanderbilt University, Nashville, TN, United States; ^5^Tennessee Center for AIDS Research, Vanderbilt University Medical Center, Nashville, TN, United States; ^6^Department of Biostatistics, Vanderbilt University Medical Center, Nashville, TN, United States; ^7^VANTAGE, Vanderbilt University Medical Center, Nashville, TN, United States; ^8^Institute for Immunology and Infectious Diseases, Murdoch University, Perth, WA, Australia; ^9^Department of Medicine, Vanderbilt University Medical Center, Nashville, TN, United States; ^10^Division of Diabetes, Endocrinology and Metabolism, Vanderbilt University, Nashville, TN, United States

**Keywords:** HIV—human immunodeficiency virus, adipose tissue, diabetes mellitus, TEMRA, T effector memory cells, GPR56, CX3CR1, memory T cells

## Abstract

Chronic T cell activation and accelerated immune senescence are hallmarks of HIV infection, which may contribute to the increased risk of cardiometabolic diseases in people living with HIV (PLWH). T lymphocytes play a central role in modulating adipose tissue inflammation and, by extension, adipocyte energy storage and release. Here, we assessed the CD4^+^ and CD8^+^ T cell profiles in the subcutaneous adipose tissue (SAT) and blood of non-diabetic (*n* = 9; fasting blood glucose [FBG] < 100 mg/dL), pre-diabetic (*n* = 8; FBG = 100–125 mg/dL) and diabetic (*n* = 9; FBG ≥ 126 mg/dL) PLWH, in addition to non- and pre-diabetic, HIV-negative controls (*n* = 8). SAT was collected by liposuction and T cells were extracted by collagenase digestion. The proportion of naïve (T_Nai_) CD45RO^−^CCR7^+^, effector memory (T_EM_) CD45RO^+^CCR7^−^, central memory (T_CM_) CD45RO^+^CCR7^+^, and effector memory revertant RA^+^(T_EMRA_) CD45RO^−^CCR7^−^ CD4^+^ and CD8^+^ T cells were measured by flow cytometry. CD4^+^ and CD8^+^ T_EM_ and T_EMRA_ were significantly enriched in SAT of PLWH compared to blood. The proportions of SAT CD4^+^ and CD8^+^ memory subsets were similar across metabolic status categories in the PLWH, but CD4^+^ T cell expression of the CD69 early-activation and tissue residence marker, particularly on T_EM_ cells, increased with progressive glucose intolerance. Use of t-distributed Stochastic Neighbor Embedding (t-SNE) identified a separate group of predominantly CD69^lo^ T_EM_ and T_EMRA_ cells co-expressing CD57, CX_3_CR1, and GPR56, which were significantly greater in diabetics compared to non-diabetics. Expression of the CX_3_CR1 and GPR56 markers indicate these T_EM_ and T_EMRA_ cells may have anti-viral specificity. Compared to HIV-negative controls, SAT from PLWH had an increased CD8:CD4 ratio, but the distribution of CD4^+^ and CD8^+^ memory subsets was similar irrespective of HIV status. Finally, whole adipose tissue from PLWH had significantly higher expression of TLR2, TLR8, and multiple chemokines potentially relevant to immune cell homing compared to HIV-negative controls with similar glucose tolerance.

## Introduction

People living with human immunodeficiency virus (HIV) are at an increased risk of developing insulin resistance and overt diabetes mellitus, but the factors contributing to the high prevalence of metabolic disease in the HIV population are not fully understood (1–3). Since HIV was identified as the cause of acquired immune deficiency syndrome (AIDS) in the early 1980s, the metabolic consequences of altered adipose tissue function in people living with HIV (PLWH) have been a major research focus (4). Early studies identified accelerated lipolysis and hepatic lipogenesis as central energy metabolism abnormalities in untreated HIV infection (5–7), while early-generation nucleoside reverse transcriptase inhibitors (NRTIs) caused adipocyte mitochondrial damage and adipose tissue fibrosis (8–12), and treatment with early protease inhibitors was accompanied by accumulation of visceral adipose tissue (VAT), hyperlipidemia, and insulin resistance (13–16). More recently, several studies describe profound changes in adipose tissue T cell populations during chronic HIV and simian immunodeficiency virus (SIV; a non-human primate virus similar to HIV) infections, which may influence adipose tissue metabolic function. These include changes in T cell surface marker phenotypes, cytokine production, antigen receptor repertoire, and capacity for latent infection with HIV or SIV provirus (17–22). Notably, several studies found that HIV and SIV were accompanied by a substantial increase in the proportion of adipose CD8^+^ T cells relative to CD4^+^ T cells, which is strikingly similar to the enrichment in CD8^+^ T cells also described in obesity (23–25).

Adipose tissue is a complex and vascularized cellular amalgam, comprised of multipotent adipocyte progenitors, mature adipocytes, fibroblasts, and immune cells of the adaptive and innate lineages. A diverse group of immune cells collectively identify and eliminate the range of viruses and other pathogens which can infiltrate adipose tissue, and these processes impact local levels of pro-inflammatory and other cytokines with subsequent effects on adipocyte regulation and energy storage and release. T lymphocytes play several beneficial and detrimental roles within this environment. Studies of obese humans and animals demonstrate an increase in adipose tissue CD8^+^ T cells and CD4^+^ T_H_1 cells, a decrease in T regulatory cells, and an increase in M1-phenotype (CD68^+^, tumor necrosis factor-α [TNF-α], interleukin [IL]-6, IL-12, and IL-23-producing) pro-inflammatory macrophages (23, 24, 26, 27) compared to non-obese controls. In animal models of obesity, the infiltration of CD8^+^ T cells into adipose tissue precedes the recruitment of macrophages (23); and the resulting increase in local IL-6, TNF-α and other inflammatory mediators act on adipocyte surface receptors and other mechanisms to inhibit insulin signaling via reduced insulin receptor substrate-1 (IRS-1), phosphoinositide 3-kinase p85α, and glucose transporter type 4 (GLUT4) expression (17, 18, 23, 28–32).

The increase in the CD8^+^ to CD4^+^ T cell ratio observed in HIV infection is like that seen in diet-induced obesity, though the mechanisms underlying the accumulation of CD8^+^ T cells in the adipose tissue of PLWH are not well-defined. Studies in macaques suggest that the high proportion of CD8^+^ T cells is not due to depletion of CD4^+^ T cells (18). Although lack of Ki-67 expression in adipose tissue T cells has been interpreted as lack of evidence for *in situ* proliferation, greater CD8^+^ TCR clonality in subcutaneous adipose tissue (SAT) implies antigen specificity might drive the increase rather than stochastic recruitment of circulating CD8^+^ T cells. This is further supported by the finding that CD8^+^ and CD4^+^ T cells in adipose tissue predominantly display a memory phenotype with increased levels of CD69 expression compared to those in blood (17, 18).

While prior studies have shown enrichment of CD8^+^ over CD4^+^ T cells in adipose tissue after HIV infection, there is a paucity of data on whether a particular subset of cells underlies this change, and whether adipose tissue T cell profiles differ according to insulin sensitivity in PLWH (as might be expected given prior findings in obesity-related insulin resistance). In this study, we hypothesized that the enrichment of CD8^+^ T cells in the adipose tissue of PLWH could be attributed to an over-representation of one or a few memory cell subtypes, and that greater CD8^+^ and CD4^+^ T cell activation would characterize the adipose tissue of diabetic PLWH. We evaluated SAT CD4^+^ and CD8^+^ T cell subsets (including naïve cells, activated cells, and central memory [T_CM_], effector memory [T_EM_], and effector memory revertant RA^+^ [T_EMRA_] cells) in PLWH vs. HIV-negative controls, and among diabetic vs. non-diabetic PLWH.

## Materials and Methods

### Study Participants

We enrolled 26 PLWH on long-term antiretroviral therapy (ART) with sustained virologic suppression from the Vanderbilt Comprehensive Care Clinic between August 2017 and June 2018. Hemoglobin A1c (HbA1c) and fasting blood glucose (FBG) were used to classify participants as non-diabetic (*n* = 9; HbA1c < 5.7% and FBG < 100 mg/dL), pre-diabetic (*n* = 8; HbA1c 5.7–6.5% and/or FBG 100–125 mg/dL), and diabetic (*n* = 9; HbA1c ≥ 6.5% and/or FBG ≥ 126 mg/dL, and on anti-diabetes medications). A group of 8 HIV-negative, non- and pre-diabetic controls were enrolled from the community. The PLWH were on ART for at least 18 months, had HIV-1 RNA <50 copies/ml for the prior 12 months, CD4^+^ count >350 cells/μl, and had no known inflammatory or rheumatologic conditions. We excluded persons with self-reported heavy alcohol use (defined as >11 drinks/week), any cocaine/amphetamine use, and those receiving corticosteroids or growth hormone.

All visits occurred in the Vanderbilt Comprehensive Care Clinic research suite or the Vanderbilt Clinical Research Center between 8 and 11 am. Participants fasted for a minimum of 8 h prior to blood collection for laboratory measurements and peripheral blood mononuclear cell (PBMC) separation (PLWH only). Blood glucose, HbA1c, high-sensitivity C-reactive protein (hsCRP), low-density lipoprotein (LDL), triglycerides, and high-density lipoprotein (HDL) were measured in the fasting blood samples at the Vanderbilt Clinical Chemistry Laboratory.

### Adipose Tissue Biopsy and T Cell Extraction

SAT biopsies were collected ~3 cm to the right of the umbilicus after anesthetizing the skin with lidocaine and infiltrating 40 ml of sterile saline and lidocaine into the subcutaneous adipose tissue as tumescent fluid. We collected ~5 grams of adipose tissue using a 2.1 mm blunt, side-ported liposuction catheter (Tulip CellFriendly™ GEMS system Miller Harvester, Tulip Medical Products) designed for the extraction of viable adipocytes and stromal vascular cells during cosmetic adipose tissue transfer procedures (33). With this approach, adipose tissue is recovered in droplets generally <3 mm in diameter, limiting the need to mechanically mince the sample, and the tissue is placed in 40–50 cc of cold saline and mixed to rinse. Any visible clots are removed before the sample is transferred to a 70 μm mesh filter for repeated saline rinses with constant stirring. The adipose tissue is then placed in a gentleMACS™ Dissociator (Miltenyi Biotec) followed by incubation with collagenase (Roche Catalog #11088866001). Mononuclear cells were separated by Ficoll-Paque Plus density gradient and cryopreserved in fetal bovine serum (FBS) with 10% DMSO.

### Flow Cytometry Analysis

PBMC and SAT mononuclear cell aliquots were processed and stained as previously published (21). In brief, matched cryopreserved PBMCs and SAT cells were quickly thawed at 37°C and suspended in R10 media (RPMI with 10% FBS). These were then washed once in phosphate buffered saline (PBS) and stained with multiple fluorescent tagged antibodies: CD3-BV786 (Clone SK7; BD Biosciences #563800), CD4-PcPCy5.5 (Clone RPA-T4; BD Biosciences #560650), CD8-A700 (Clone PRA-T8; BD Biosciences #557945), CD57-FITC (Lot 4182924; BD Pharmingen # 555619), CX_3_CR1-PE (Clone 2A9-1; BD Biosciences #565796), CD45RO-PECF594 (Clone UCHL1; BD Biosciences #562299), CD14-V500 (Clone M5E2, BD Biosciences #561391), CD19-V500 (Clone HIB19; BD Biosciences #561121), LIVE/DEAD Fixable Aqua (ThermoFisher; L34957), CD69-APC (Clone FN50; BD Biosciences #560711), CCR7-V450 (Clone 150503; BD Biosciences #562555), GPR56-PECy7 (Clone CG4; BioLegend #358205), and HLA-DR APC Cy7 (Clone G46.6; BD Biosciences #561358). CCR7 and CX_3_CR1 antibody stains were performed at 37°C; the remainder was stained at room temperature. Cells were analyzed on a 4-laser FACSAria III (all samples from PLWH) or 5-laser LSRII (HIV-negative SAT samples) (BD Biosciences, San Jose, CA). Bead calibration was used to standardize runs done on different days. Flow cytometry data were analyzed using FlowJo software (version 10.4.1) and Cytobank (version 6.3.1) (34, 35).

**Figure 1:**
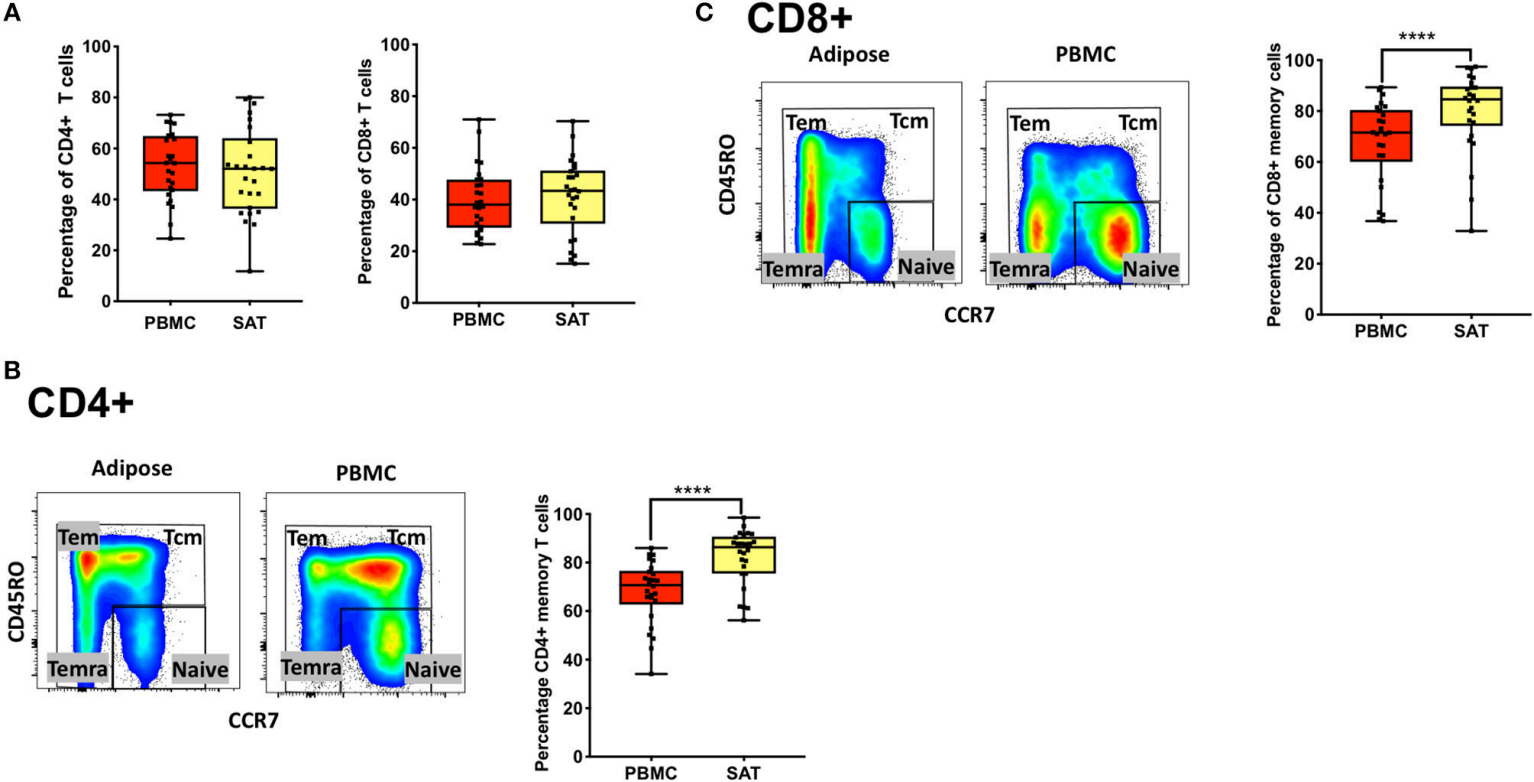
Subcutaneous adipose tissue from PLWH has a higher percentage of CD4^+^ and CD8^+^ memory T cells compared to matched blood samples. **(A)** Frequencies of total CD4^+^ and CD8^+^ T cells (percentage of CD3^+^ cells) in subcutaneous adipose tissue (SAT) and blood (PBMC) from all 26 PLWH. **(B)** Representative plot showing gating of CD4^+^ memory T cells (gated on CD3^+^) in SAT and PBMC depicted by the yellow and red shading, respectively; on the right are individual values and means. **(C)** Representative plot showing total CD8^+^ memory T cells in adipose tissue and PBMC. The box and whiskers plot indicate mean ± SD. Wilcoxon matched pair signed test was used to calculate statistics (A-C); ^****^p < 0.0001.

The T cell memory populations in our study are defined as naive T cells (T_Nai_, CD45RO^−^CCR7^+^), T effector memory (T_EM_, CD45RO^+^CCR7^−^), T central memory (T_CM_, CD45RO^+^CCR7^+^), and T effector memory revertant/re-expressing CD45-RA (T_EMRA_, CD45RO^−^CCR7^−^). Representative gating strategies in Supplementary Figure 1 and Figure 1 show the phenotypic markers used to define the memory subsets (CD45RO and CCR7). Total memory cells are a combined group consisting of T_EM_, T_CM_, and T_EMRA_.

### Adipose Tissue Gene Expression

Adipose tissue was rinsed, placed in cryovials and snap-frozen in liquid nitrogen immediately after collection for subsequent mRNA expression assays. mRNA was extracted after mechanical lysis with the Qiagen RNeasy Lipid Tissue Kit. We used the Nanostring nCounter Plex^2^ human inflammation panel to quantify mRNA expression of over 250 genes spanning a broad range of relevant immune pathways including interleukin signaling, Ras, T-cell markers, and Toll-like receptor signaling. Adipose tissue mRNA expression levels were normalized using 14 synthetic spike-ins (6 positive controls and 8 negative controls) and 6 cellular housekeeping genes included in the assay (GAPDH, GUSB, HPRT1, PGK1, TUBB, and CLTC). We first calculated the coefficient of variation (CV) for the control genes. The CV of the positive controls is proportional to the technical variability introduced by the nCounter platform. The CV for the housekeeping controls is proportional to the confounding biological variation due to sample input. The mean endogenous CV shows the global noise of experimentally observed genes. After evaluating different normalization approaches based on CV values, we developed a normalization strategy including the following steps. First the background count levels were calculated using the mean of negative controls, then subtracted from each sample. The normalization factor for sample/RNA content was calculated using the geometric mean of a set of pre-specified annotated housekeeping genes. The algorithm normalizes for sample or RNA content, i.e., “pipetting” fluctuations, using the geometric mean of pre-specified annotated housekeeping genes. The count data were then divided by the normalization factor to generate counts normalized to the geometric mean of housekeeping genes. None of the housekeeping genes differed significantly in their distribution between study groups.

### Statistical Analyses

For comparisons, PLWH were grouped according to metabolic status as non-diabetic (*n* = 9), pre-diabetic (*n* = 8), and diabetic (*n* = 9). The 8 HIV-negative controls were matched to 9 PLWH with similar HbA1c and body mass index (BMI) values to yield a similar comparison group (one control was matched to two PLWH). Percentages of CD4^+^ and CD8^+^ subsets were compared between paired blood and adipose tissue samples using Wilcoxon signed-rank tests for paired data. Differences between the PLWH metabolic groups, and between HIV-negative controls and the PLWH, were calculated using Mann-Whitney and Kruskal Wallace tests. When significant between-group comparisons were noted among the PLWH, univariable and multivariable linear regression were used to assess the relationship of cellular populations with progressive glucose intolerance. Statistical analyses and graphs were performed using SPSS (IBM, Armonk, NY), R (www.r-project.org), and GraphPad Prism 7 (GraphPad Software, La Jolla, CA),

Adipose tissue genes were grouped according to immune system pathways specified in the NanoString kit, and normalized mRNA levels were compared between the PLWH and HIV-negative participants. DEseq2 was used to detect differential expression between two groups based on the normalized count data, taking into account technical and biological variability (36, 37). Differences in gene expression were calculated as fold-changes, and *p*-values were adjusted for multiple comparisons using the Benjamini-Hochberg procedure. Analyses were performed using R. Volcano plots displaying the NanoString data were generated using XL-STAT.

## Results

### Clinical and Demographic Characteristics of PLWH and HIV-Negative Subjects

The non-diabetic (*n* = 9), pre-diabetic (*n* = 8), and diabetic (*n* = 9) groups of PLWH are compared in Table 1. Age, race and sex were similar across the groups, as were CD4^+^ nadir, duration of ART, and the proportions receiving integrase inhibitor-based regimens (*p* > 0.05 for all comparisons). BMI and waist circumference increased with progressive glucose intolerance (*p* = 0.04 for both). The HIV-negative controls (*n* = 8) and comparator PLWH (9 out of 26 total) were similar in age, BMI, and HbA1c values (*p* > 0.05 for all), though the controls were more likely to be female and white (Table 2). Bold values in Tables 1, 2 indicate *p* < 0.05.

**Table 1:** Cohort demographic and clinical characteristics according to glucose tolerance.

**Variable**	**HIV (+) Non-diabetic** **HbA1c < 5.7 *and* FG < 100 mg/dL** **(*n* = 9)**	**HIV (+) Pre-diabetic** **HbA1c 5.7–6.5 *or* FG = 100–125 mg/dL** **(*n* = 8)**	**HIV (+) Diabetic on** **pharmacotherapy** **(*n* = 9)**	***P*-value**
Age, median yr (IQR)	39 (38, 49)	59 (33, 63)	54 (45, 61)	0.22
Female (%)	1 (11%)	2 (25%)	4 (44%)	0.23
Caucasian race (%)	4 (40%)	4 (50%)	3 (33%)	0.78
BMI, kg/m^2^	30.8 (27.8, 36.4)	32.7 (30.8, 37.7)	40.2 (34.0, 51.9)	**0.04**
Waist: hip ratio	0.97 (0.92, 1.00)	0.97 (0.92, 1.04)	0.98 (0.96, 1.09)	0.60
Waist Circumference, cm	106 (101, 114.5)	107 (101, 111)	116 (110, 142)	**0.04**
**GLUCOSE METABOLISM MEASUREMENTS**				
Hemoglobin A1c, %	5.3 (5.1, 5.4)	5.6 (4.9, 5.9)	7.2 (6.1, 9.8)	**<0.001**
Fasting glucose, mg/dL	87 (75, 96)	116 (102, 122)	141 (120, 238)	**<0.001**
**PLASMA LIPIDS**				
Cholesterol, mg/dL	181 (149, 194)	185 (172, 210)	175 (143, 193)	0.28
LDL, mg/dL	95 (77, 109)	116 (96, 131)	82 (56, 107)	**0.05**
Fasting HDL, mg/dL	52 (39, 81)	38 (34, 56)	43 (35, 56)	0.31
Fasting TG, mg/dL	104 (61, 173)	196 (82, 257)	149 (114, 267)	0.37
**IMMUNE MARKERS**				
High sensitive C-reactive protein, mg/dl	2.7 (1.1, 8.4)	3.1 (0.9, 4.2)	3.1 (1.9, 9.8)	0.67
CD4 at study visit	803 (767, 1065)	702 (643, 942)	1056 (950, 1167)	**0.02**
CD4 at start of therapy	482 (364, 617)	448 (154, 567)	323 (173, 432)	0.12
CD4:CD8 ratio	1.07 (0.88, 1.43)	1.04 (0.73, 1.18)	1.36 (0.94, 1.52)	0.49
**ANTIRETROVIRAL THERAPY HISTORY**				
Exposure to AZT, *n* (%)	2 (22%)	3 (38%)	5 (56%)	0.35
Duration of ART therapy, yr	7.5 (4.0, 13.2)	11.8 (5.8, 18.9)	11.6 (5.4, 18.6)	0.56
Integrase inhibitor-based therapy	8 (89%)	4 (40%)	7 (78%)	0.18
**OTHER**				
Smoker (%)	2 (22%)	2 (25%)	2 (22%)	0.99
Hepatitis C (%)	1 (11%)	0 (0%)	2 (22%)	0.36

*ART, antiretrovirals; AZT, azidothymidine; BMI, body mass index; FG, fasting glucose; HDL, high-density lipoproteins; IQR, interquartile range; LDL, low-density lipoproteins; TG, triglycerides; yr, year*.

*The bolded values are p-values < 0.05, indicating statistical significance*.

**Table 2:** Comparison of HIV-negative participants and comparator non- and pre-diabetic PLWH.

**Variable**	**PLWH** **(*n* = 9)**	**HIV-negative controls** **(*n* = 8)**	***P*-value**
Age, median years (IQR)	39 (38, 49)	49 (37, 62)	0.32
Female (%)	1 (10%)	5 (63)	**0.02**
Caucasian race (%)	4 (40%)	8 (100%)	**<0.01**
BMI, kg/m^2^	30.8 (27.8, 36.4)	39.35 (33.3, 48.4)	0.89
**GLUCOSE METABOLISM MEASUREMENTS**			
Hemoglobin A1c, %	5.3 (5.15, 5.4)	5.7 (5.4, 5.9)	0.84

*The bolded values are p-values <0.05, indicating statistical significance*.

### Adipose Tissue From PLWH Is Enriched in Memory CD8^+^ and CD4^+^ T Cells

We found no difference in the percentage of total CD8^+^ and CD4^+^ T cells (gated on CD3^+^ T cells) between SAT and peripheral blood from PLWH Figure 1A. We also observed, as previously described by other groups, a higher percentage of total CD4^+^ memory T cells (Figure 1B) and CD8^+^ memory T cells (Figure 1C) in SAT compared to peripheral blood (*p* < 0.0001) (17).

### SAT From PLWH Is Enriched in CD4^+^ and CD8^+^ T_EM_ and T_EMRA_ Cells Compared to Blood

Despite the growing body of literature on adipose tissue immune cells, the distribution of memory T cell subsets in adipose tissue from PLWH has not been characterized, and the role of memory T cell subsets within tissue compartments in general is not well-understood. A prior study assessed the distribution of CD4^+^ and CD8^+^ T_Nai_, T_EM_, T_CM_, and T_EMRA_ subsets in lung, spleen, colon, ileum, jejunum, and lymph nodes, but not adipose tissue, from healthy donors, and found that CD4^+^ T_Nai_, T_EM_, and T_CM_ proportions in lung and pulmonary and mesenteric lymph nodes were similar to the proportion in blood, while jejunum, ileum, and colon were roughly 3- to 4-fold enriched in T_EM_ (38). In all tissues, CD4^+^ T_EMRA_ were relatively sparse (<10%). In contrast, ~30% of CD8^+^ T cells in blood, spleen, and lung had a T_EMRA_ phenotype, while the CD8^+^ T cells in jejunum, ileum, and colon were overwhelmingly T_EM_ (38). A subsequent study in the adipose tissue of mice found approximately equal proportions of CD4^+^ T_EM_ and CD4^+^ T_RM_ cells (a “resident memory” phenotype defined by the authors as CD69 expression on T_EM_ cells) in adipose tissue samples, and fewer T_CM_ (39). However, CD8^+^ memory cells were predominantly T_RM_ in SAT, with similar, lower proportions of T_EM_ and T_CM_. The same study looked at mesenteric adipose tissue in macaques and again found high levels of CD8^+^ T_RM_, while CD4^+^ cells were primarily T_Nai_ and T_CM_, with low levels (<10%) of T_EM_ and T_RM_, potentially highlighting important differences between species.

**Figure 2:**
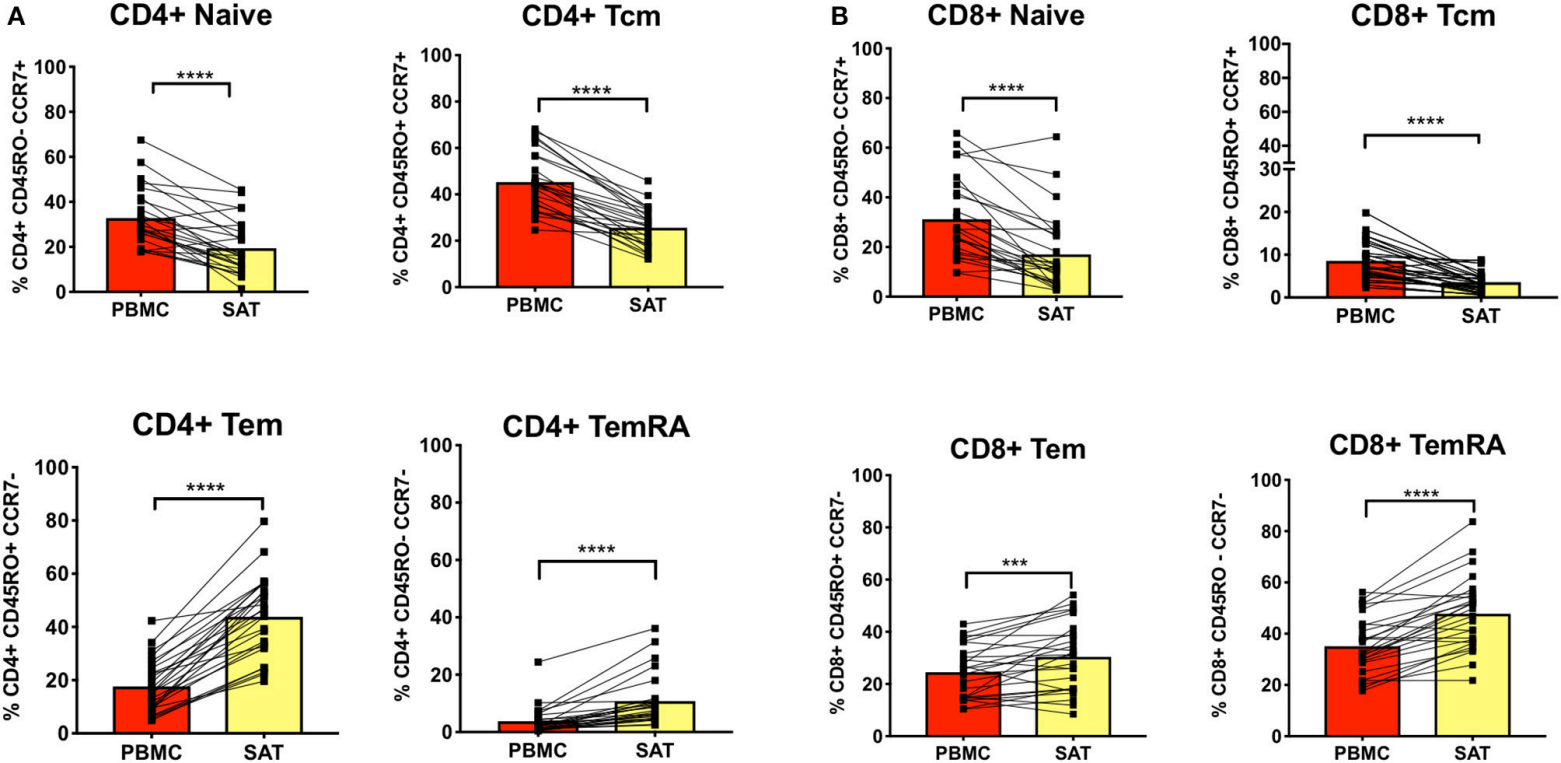
Subcutaneous adipose tissue has a higher percentage of T_EM_ (CD45RO^+^ CCR7^−^) and T_EMRA_ (CD45RO^−^ CCR7^−^) cells compared to blood in PLWH. **(A)** The bar graphs show frequencies of CD4^+^ T_Nai_, T_EM_, T_CM_, and T_EMRA_ cells in subcutaneous adipose tissue (SAT) and blood (PBMC) from all twenty-six subjects. **(B)** Frequencies of CD8^+^ T_Nai_, T_EM_, T_CM_, and T_EMRA_ cells in SAT and PBMC. The numbers in the bar graphs on the left indicate mean and diagonal lines indicate matched pairs of SAT and PBMCs. Wilcoxon matched pair signed test was used to calculate statistics ^****^p < 0.0001; ^***^p < 0.001.

We first assessed CD4^+^ and CD8^+^ memory subsets in SAT and blood from all 26 PLWH. Multiparameter gates were used to quantify memory CD4^+^ and CD8^+^ T cells within each sample as shown in Supplementary Figures 1, 9. Each sample had >100,000 ungated cells (Supplementary Figures 10A–C). SAT was significantly enriched in CD4^+^ and CD8^+^ T_EM_ and T_EMRA_ cells (Figures 2A,B) compared to blood but had fewer T_Nai_ and T_CM_ cells. We then compared the average T_Nai_, T_CM_, T_EM_, and T_EMRA_ fractions within SAT and blood among the 26 PLWH (Supplementary Figure 2). We found that CD4^+^ T cells in SAT can be ranked by frequency as T_EM_ > T_CM_ > T_Nai_ > T_EMRA_, whereas peripheral blood contains CD4^+^ T_CM_ > T_Nai_ > T_EM_ > T_EMRA_. In contrast, SAT CD8^+^ T cells are primarily T_EMRA_ followed by T_EM_.

### The Relative Distribution of SAT Memory T Cell Subsets Does Not Differ With Metabolic Status in PLWH

The study of adipose tissue T cells in mice by Han et al. also found that recall responses of adipose tissue memory T cells were enhanced after antigen re-challenge, with downregulation of several metabolic pathways in whole adipose tissue (including lipid biosynthesis and cholesterol and long-chain fatty-acyl-CoA metabolic processes) and a detectable reduction in serum levels of adiponectin and cholesterol (39). These findings suggest a possible mechanism by which the accumulation, and subsequent stimulation, of memory cells in adipose may disrupt metabolic homeostasis.

In light of prior animal studies indicating a potential contribution of effector memory T cells to inflammation and impaired metabolic function in adipose tissue, we sought to characterize the distribution of SAT memory T cell subsets in PLWH to determine if there were clear differences between non-diabetics, pre-diabetics, and diabetics in the relative proportions of T_Nai_, T_CM_, T_EM_, and T_EMRA_ cells. We utilized t-distributed Stochastic Neighbor Embedding (t-SNE) to visualize groups of adipose tissue and blood CD4^+^ and CD8^+^ T cell populations based on 12-color flow cytometry staining. Supplementary Figure 3 shows the distribution of T_Nai_, T_CM_, T_EM_, and T_EMRA_ cells in CD4^+^ and CD8^+^ T cells in the adipose tissue and blood from four representative non-diabetic and diabetic PLWH. The plots showed fewer T_Nai_ cells and enriched CD4^+^ and CD8^+^ T_EM_ and T_EMRA_ cell fractions in the SAT compared to the matched peripheral blood.

**Figure 3:**
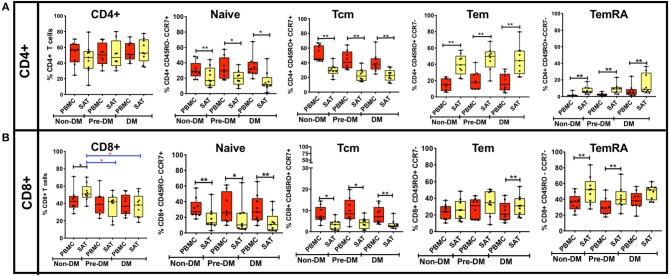
Analysis of CD4^+^ and CD8^+^ memory subsets by metabolic status in PLWH. **(A)** The bar graphs on the top row show frequencies of CD4^+^ T_Nai_, T_EM_, T_CM_, and T_EMRA_ cells in subcutaneous adipose tissue (SAT) and blood (PBMC) from all twenty six PLWH. Participants are grouped based on glucose tolerance: non-diabetic (Non-DM): hemoglobin A1c < 5.7% and fasting glucose < 100 mg/dL (*n* = 9); Pre-DM: hemoglobin A1c 5.7–6.4% or fasting glucose 100–125 mg/dL (*n* = 8); DM: hemoglobin A1c > 6.5% and/or fasting glucose > 126 mg/dL, and on anti-diabetes medication (*n* = 9). **(B)** Frequencies of CD8^+^ T_Nai_, T_EM_, T_CM_, and T_EMRA_ cells in SAT and PBMC. The box and whiskers plot indicate mean ± SD. Wilcoxon matched pair signed test was used to calculate statistics between matched PBMC and SAT T cells, Mann-Whitney test was used to analyze differences in SAT and PBMC T cell subsets between metabolic groups; ^**^p < 0.01, ^*^p < 0.05.

The t-SNE findings were congruous with our results from 2-dimensional flow cytometry gating. For CD4^+^ T cells, the proportion of T_Nai_ and T_CM_ cells were significantly lower compared to blood in all three groups, while the proportion of T_EM_ and T_EMRA_ cells was significantly higher (Figure 3A). However, none of the adipose memory subsets in the pre-diabetics or diabetics were significantly different from the non-diabetics in pairwise comparisons. There was less consistency in the relative proportions of CD8^+^ memory cells in the blood and adipose tissue. While CD8^+^ T_Nai_ and T_CM_ cells were significantly lower in the SAT in all three groups (Figure 3B), SAT CD8^+^ T_EM_ cells were only significantly higher compared to blood in the diabetics, and SAT T_EMRA_ cells were only significantly higher in the non- and pre-diabetic groups. In summary, these findings suggest that while adipose tissue is enriched in CD4^+^ and CD8^+^ T_EM_ and T_EMRA_ cells compared to blood, the relative distribution of naïve and memory T cells within SAT does not markedly vary by metabolic status in PLWH.

Next, we assessed SAT memory T cells according to BMI category and age (Supplementary Figures 4, 5). We observed no significant differences in SAT CD4^+^ and CD8^+^ memory T cell populations according to BMI status. When we compared naïve and memory T cell subsets by age, we found the proportion of CD4^+^ T_Nai_ cells in SAT from PLWH <35 years (34% of total CD4^+^ cells) was significantly higher than in older subjects 35–55 years (12%, *p* < 0.05) and those >55 years (16%, *p* < 0.01). The relative reduction in CD4^+^ T_Nai_ cells appeared principally due to an increase in the proportions of T_EM_ cells at higher ages; T_EM_ cells constituted 29% of total CD4^+^ cells in participants <35 years, but rose to 50% in those 35–55 years (*p* = 0.06) and 47% in those >55 years (*p* < 0.01). These differences were less pronounced for CD8^+^ T cells; the T_EM_ proportion in PLWH >55 years was significantly higher than those <35 years (38% vs. 22%, *p* < 0.05), but the difference was not significant for those ages 35–55. Our findings were similar to a prior study of health donors, which found the proportion of total CD4^+^ memory (CD45RO^+^) T cells in lung and mesenteric lymph nodes, spleen, ileum and colon also rose with increasing age (38).

### CD4^+^ T Cell CD69 Expression Increases With Progressive Glucose Intolerance in PLWH

CD69 is an inducible, early-activation indicator which also serves as a putative tissue-resident marker on memory T cells in human, as well as in animal, mucosal and lymphoid tissues (38, 40, 41), but is largely absent on memory T cells in blood (38). CD69 has been used as a marker of adipose tissue resident memory cells in animals (39), including in SIV-infected macaques (18), as well as in prior studies of PLWH (17, 19). At present, there are few studies on CD69 expression on adipose tissue T cells in PLWH, none of which has looked at their link with metabolic status.

**Figure 4:**
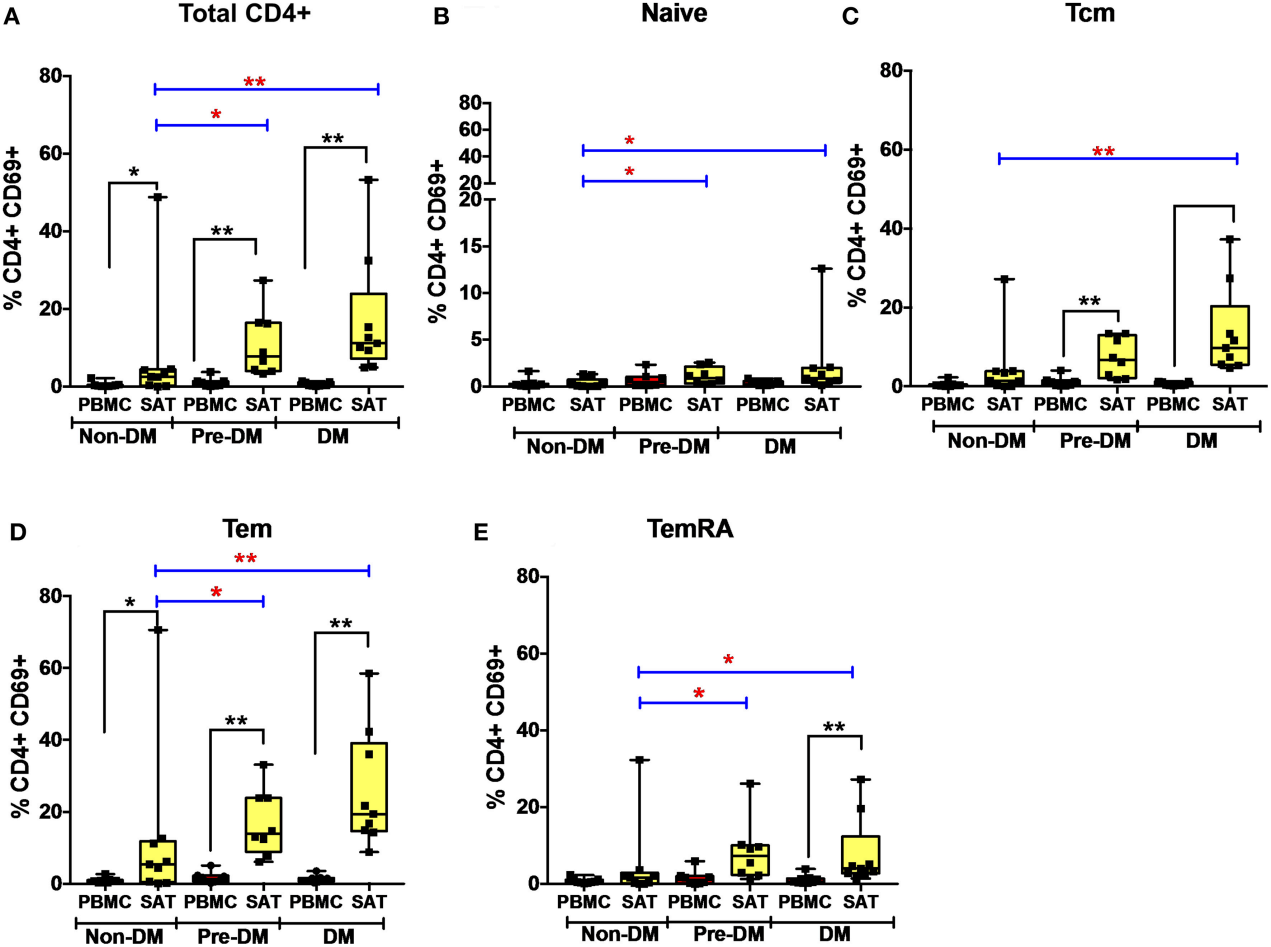
CD69 expression on subcutaneous adipose tissue CD4^+^ T cells increases with progressive glucose intolerance. **(A–E)** Frequencies of CD4^+^ total T cells, T_Nai_, T_CM_, T_EM_, and T_EMRA_ cells expressing the CD69 activation and putative tissue-residence marker in subcutaneous adipose tissue (SAT) and blood (PBMC). The box and whiskers plot indicate mean ± SD. Wilcoxon matched-pair rank test was used to calculate differences between PBMC and SAT. Mann-Whitney test used to calculate differences between groups; blue lines and red ^*^ depict differences between groups ^**^p < 0.01, ^*^p < 0.05.

We measured CD69 expression on memory T cell subsets in SAT and blood (gating strategy shown in Supplementary Figure 6). As reported in prior studies of PLWH, CD69 expression was present on CD4^+^ T cell subsets from SAT but almost absent in peripheral blood (Figure 4) (17, 19), whereas CD69 expression on CD8^+^ T cells from SAT and blood was similar (Supplementary Figure 11A). While we observed the relative proportions of SAT CD4^+^ and CD8^+^ T_CM_, T_EM_, and T_EMRA_ cells to be similar regardless of metabolic status in PLWH (Figure 3), expression of CD69 on CD4^+^ T cells rose with progressive glucose intolerance in a step-wise progression from non-diabetic, to pre-diabetic, to diabetic. Compared to non-diabetics, diabetic PLWH had significantly higher CD69 expression on total CD4^+^ T cells, T_CM_, and T_EM_ (*p* < 0.01 for all three), as well as T_Nai_ and T_EMRA_ (*p* < 0.05 for both, Figure 4). Similarly, the pre-diabetics also had higher CD69 expression on SAT total CD4^+^ T cells and T_Nai_, T_EM_, and T_EMRA_ (*p* < 0.05 for all) compared to non-diabetics, but not on T_CM_ (*p* < 0.07). In contrast, we did not observe any significant differences in CD69 expression on CD8^+^ T cells according to metabolic status.

Given the stepwise progression of CD69 expression on CD4^+^ T cell subsets with rising glucose intolerance, and the significant pairwise comparisons, a linear regression model was used to assess CD69 expression according to metabolic status. Mean CD4^+^ T cell CD69 expression for non-diabetics, pre-diabetics, and diabetics is shown in Table 3. Frequencies of CD69^+^ cells were natural-log transformed to improve normality (Shapiro-Wilk *p* < 0.01 for all untransformed CD4^+^ and CD8^+^ T cell subsets). Expression of CD69 on total CD4^+^ T cells rose with progressive glucose intolerance (Table 3, *p* = 0.004), which was robust to adjustment for BMI (*p* = 0.03 for metabolic status) and to age (*p* = 0.01 for metabolic status) in separate models (data not shown). Among CD4^+^ T cell subsets, progression from non-diabetic to diabetic groups was accompanied by increased CD69 expression on T_CM_ (*p* = 0.02), T_EM_ (*p* = 0.04), and T_EMRA_ (*p* = 0.04) cells.

**Table 3:** Mean SAT CD4^+^ and CD8^+^ T cell CD69 expression according to glucose tolerance.

**T cell subset**	**Non-diabetic** ***n* = 9**	**Pre-diabetic** ***n* = 8**	**Diabetic** ***n* = 9**	*p*-Value^*^
Total CD4^+^ T cells	7.5%	10.9%	17.2%	**0.004**
CD4 T_NAIVE_	0.6%	1.1%	2.0%	**0.03**
CD4 T_CENTRAL MEMORY_	5.9%	7.8%	11.9%	**0.02**
CD4 T_EFFECTOR MEMORY_	14.7%	18.2%	22.3%	**0.04**
CD4 ${\text{T}}_{\text{EFFECTOR MEMORY R}{\text{A}}^{+}}$	4.9%	8.9%	7.3%	**0.04**
Total CD8^+^ T cells	5.4%	6.2%	10.0%	0.09
T_NAIVE_	1.5%	0.6%	4.2%	0.28
T_CENTRAL MEMORY_	6.9%	5.5%	12.8%	0.45
T_EFFECTOR MEMORY_	11.9%	9.3%	17.0%	0.19
${\text{T}}_{\text{EFFECTOR MEMORY R}{\text{A}}^{+}}$	5.0%	5.7%	9.0%	0.12

* *Univariable linear regression. The association of total CD4^+^ T cell CD69 expression with progressive glucose intolerance was robust to adjustment for BMI (p = 0.03) and to age (p = 0.01) in separate models*.

*The bolded values are p-values <0.05, indicating statistical significance*.

### CD57 Expression Is Higher on SAT CD4^+^ and CD8^+^ T Cells, but Does Not Vary With Metabolic Status in PLWH

We previously reported a higher proportion of late-differentiated, CD57^+^ CD8^+^ T cells in the SAT of non-diabetic PLWH compared to blood (37 vs. 23%, *p* < 0.01) (21). CD57 is a terminally-sulfated glycan carbohydrate epitope found on T cells and natural killer (NK) cells which serves as a marker of late differentiation, though there is limited consensus as to whether CD57 is a marker of an inability to proliferate in response to antigen stimulation, signifies reduced replicative capacity, or represents an increased susceptibility to activation-induced apoptosis (42–44). Prior studies have shown that CD57 expression on CD4^+^ and CD8^+^ T cells is higher in the blood of PLWH compared to that of HIV-negative controls (44–46). CD8^+^ T cells expressing CD57 produce more interferon-γ and TNF-α after TCR stimulation than CD57^−^ T cells, and CD57^+^ CD8^+^ T cells have a distinct gene expression profile characterized by greater cytotoxic effector potential (e.g., production of perforin, granzymes, and granulysin) (47, 48). Additionally, a higher percentage of CD57^+^ expression on T cells has been implicated in other inflammatory diseases, such as rheumatoid arthritis (49) and beryllium-induced disease (50).

Given the potential pro-inflammatory effects of CD57^+^ T cells in adipose tissue, we compared CD4^+^ and CD8^+^ T cell expression of CD57 in SAT vs. blood, and according to metabolic status (Supplementary Figure 11). In all three metabolic groups, expression of CD57 on SAT CD8^+^ T cells was higher compared to blood, confirming our prior study findings (21). Among the memory subsets, CD57 expression was highest on CD4^+^ T_EMRA_ and CD8^+^ T_EM_ and T_EMRA_ cells. However, we did not observe an increase in CD57 expression on either CD4^+^ or CD8^+^ memory cell subsets with progressive glucose intolerance, with the exception of higher CD57 expression on CD4^+^ T_CM_ in diabetic individuals compared to those without diabetes.

### CD4^+^CD69^lo^ Cells Co-expressing CD57, CX_3_CR1, and GPR56 Are Associated With Increasing Glucose Intolerance

Adipose tissue serves as a reservoir for both latently HIV-infected CD4^+^ T cells and free HIV RNA virus, and in the Genotype-Tissue Expression (GTEx) project adipose tissue contained one of the highest levels of CMV transcripts (17, 18, 21, 51). These findings suggest adipose tissue may also serve as a site for anti-viral immune activity. Recent studies of CD4^+^ T_EMRA_ cells have identified major subsets based on G protein-coupled receptor GPR56 expression, with virus-specific cells more frequently GPR56^+^ and more clonally expanded compared to GPR56^−^ cells (52). Increased expression of GPR56 and killer-like receptors (KLR) has been linked to higher cytokine expression by memory CD4^+^ T cells, including T cells obtained from liver tissue (53). CD4^+^ and CD8^+^ T_EMRA_ cytotoxic T cells expressing GPR56 have also been associated with increased co-expression of CX_3_CR1 (52, 54, 55). CX_3_CR1 receptor is expressed on terminally differentiated T cells, gamma-delta T cells and NK cells, and has also been identified as a marker of anti-CMV T cells (55–58).

**Figure 5:**
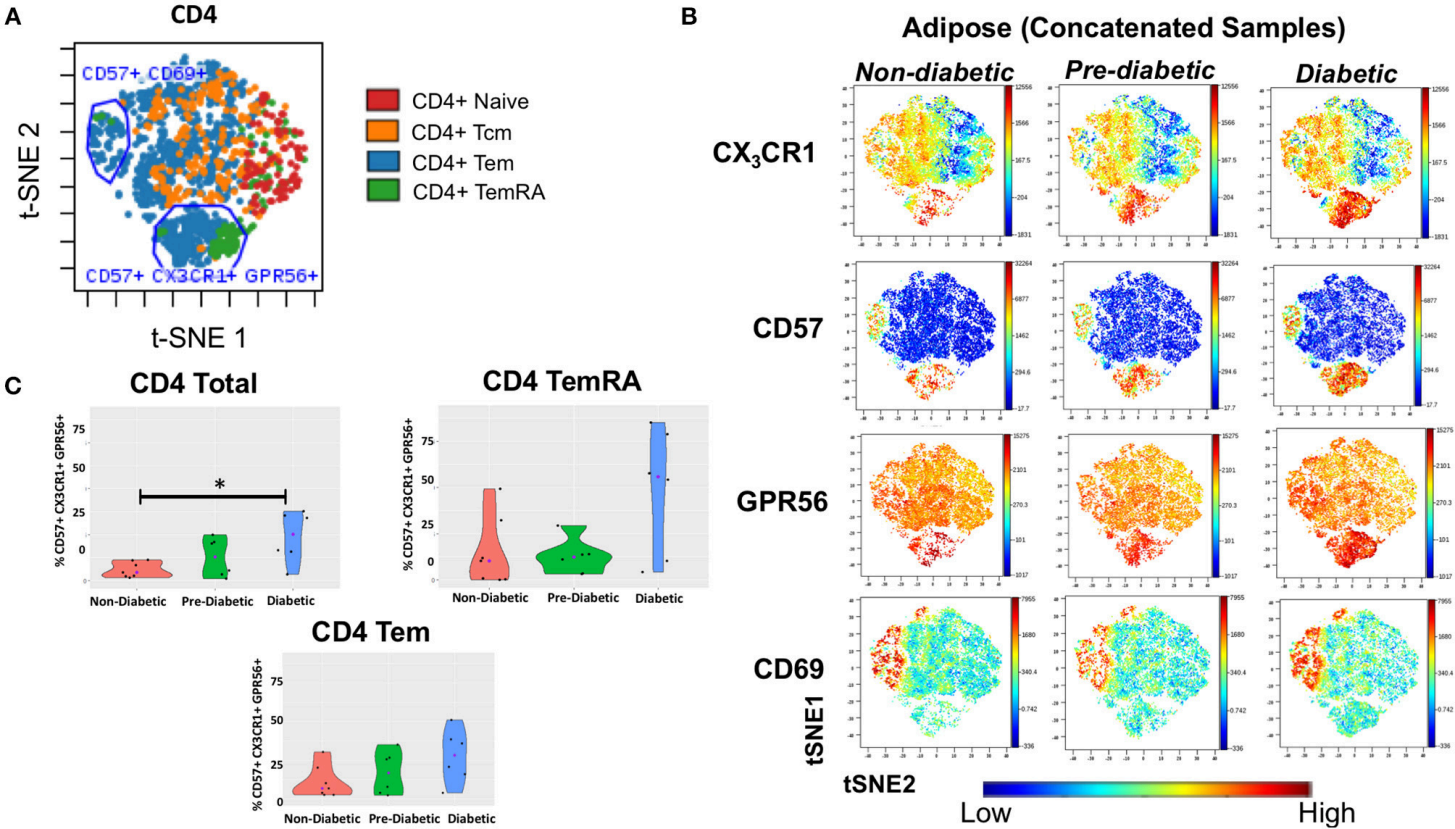
CD4^+^ T cells co-expressing CD57, CX_3_CR1, GPR56 in subcutaneous adipose tissue increase with progressive glucose intolerance. viSNE plots were generated in cytobank to identify groups of cells that differ between non-diabetic, pre-diabetic and diabetic categories. **(A)** Clusters of CD4^+^, T_Nai_, T_EM_, T_CM_, and T_EMRA_ cells from subcutaneous adipose tissue (SAT). **(B)** Concatenated viSNE plots of non-diabetic, pre-diabetic, and diabetic PLWH showing clusters of cells expressing CX_3_CR1, CD57, GPR56, and CD69. **(C)** Violin plots showing percentage of total CD4^+^, T_EM_, and T_EMRA_ cells co-expressing CD57, CX_3_CR1, and GPR56. Mann-Whitney test used to analyze differences between metabolic groups; ^*^P < 0.05.

To assess the presence of GPR56^+^ CX_3_CR1^+^ T cells, and the parent memory cell population(s), we used t-SNE and viSNE to identify surface marker clusters that differed between non-diabetic, pre-diabetic, and diabetic individuals. We identified a group of cells that expressed CD57, lacked CD69, and also co-expressed GPR56 and CX_3_CR1. A representative plot of t-SNE maps generated from CD4^+^ gated T cells showed that the CD57^+^ CX_3_CR1^+^ GPR56^+^ co-expression was mainly on T_EM_ and T_EMRA_ cells (Figure 5A). Concatenated viSNE plots of non-diabetic, pre-diabetic and diabetic PLWH demonstrated two distinct clusters of cells: CD57^−/+^ CD69^+^ CX_3_CR1^−/+^ GPR56^−/+^ and CD57^+^ CD69^lo^ CX_3_CR1^+^ GPR56^+^ (Figure 5B). A significantly larger proportion of total CD4^+^ and CD4^+^ T_EMRA_ cells in SAT from diabetics were CD57^+^ CD69^lo^ CX_3_CR1^+^ GPR56^+^ compared to SAT from non-diabetics (*p* = 0.051), and approached significance for CD4^+^ T_EM_ cells (*p* = 0.07) (Figure 5C). Of note, we observed a similar population of CD4+ T_EMRA_ and T_EM_ cells co-expressing CD57^+^ CX_3_CR1^+^ GPR56^+^ in matched PBMC samples which, again, increased with glucose intolerance and were significantly different between diabetic and non-diabetic PLWH (total CD4^+^ [*p* < 0.01], CD4^+^ T_EM_ [*p* = 0.05] and CD4^+^ T_EMRA_ [*p* < 0.05]) (Supplementary Figure 6). Similar plots of CD8^+^ T cells were generated for adipose tissue (Supplementary Figure 7) and PBMC (data not shown) from PLWH. As with the CD4^+^ T cells, CD57^+^ CX_3_CR1^+^ GPR56^+^ co-expressing CD8^+^ T cells were predominantly T_EM_ and T_EMRA_, though there were no significant differences between non-diabetics and diabetics.

Lastly, we assessed CD57^+^ CX_3_CR1^+^ GPR56^+^ co-expression on SAT CD4^+^ and CD8^+^ T cells from our HIV-negative controls (Supplementary Figure 8). We identified two distinct clusters of CD4^+^ and CD8^+^ T_EM_ and T_EMRA_ cells co-expressing CD57, CX_3_CR1, and GPR56 though the expression was more diffuse compared to SAT cells from PLWH, particularly for GPR56.

Taken together, our results demonstrate a population of CD57^+^CX_3_CR1^+^GPR56^+^ co-expressing predominantly CD4^+^ T_EMRA_ cells in both blood and SAT of PLWH which appear to be associated with diabetes. The expression of the GPR56 and CX_3_CR1 markers may indicate these cells are virus-specific TEMRA cells, a population that could contribute to inflammation in adipose tissue.

### SAT CD4^+^ and CD8^+^ T Cell Memory Subsets Compared by HIV Status

Couturier et al. identified a major shift in the CD4:CD8 T cell ratio in PLWH compared to HIV-negative controls (17), a finding that has been replicated in subsequent HIV and SIV studies (18, 19, 21). Specifically, adipose tissue stromal vascular fraction (SVF) CD3^+^ T cells from individuals without HIV were predominantly memory CD4^+^ CD45RO^+^ T cells rather than memory CD8^+^ T cells. In contrast, this distribution was reversed in PLWH, with more memory CD8^+^ T cells in the adipose tissue, which represented an ~50% enrichment in memory CD8^+^ T cells over the peripheral blood, and could not be attributed to differences in peripheral blood T cell subsets

**Figure 6:**
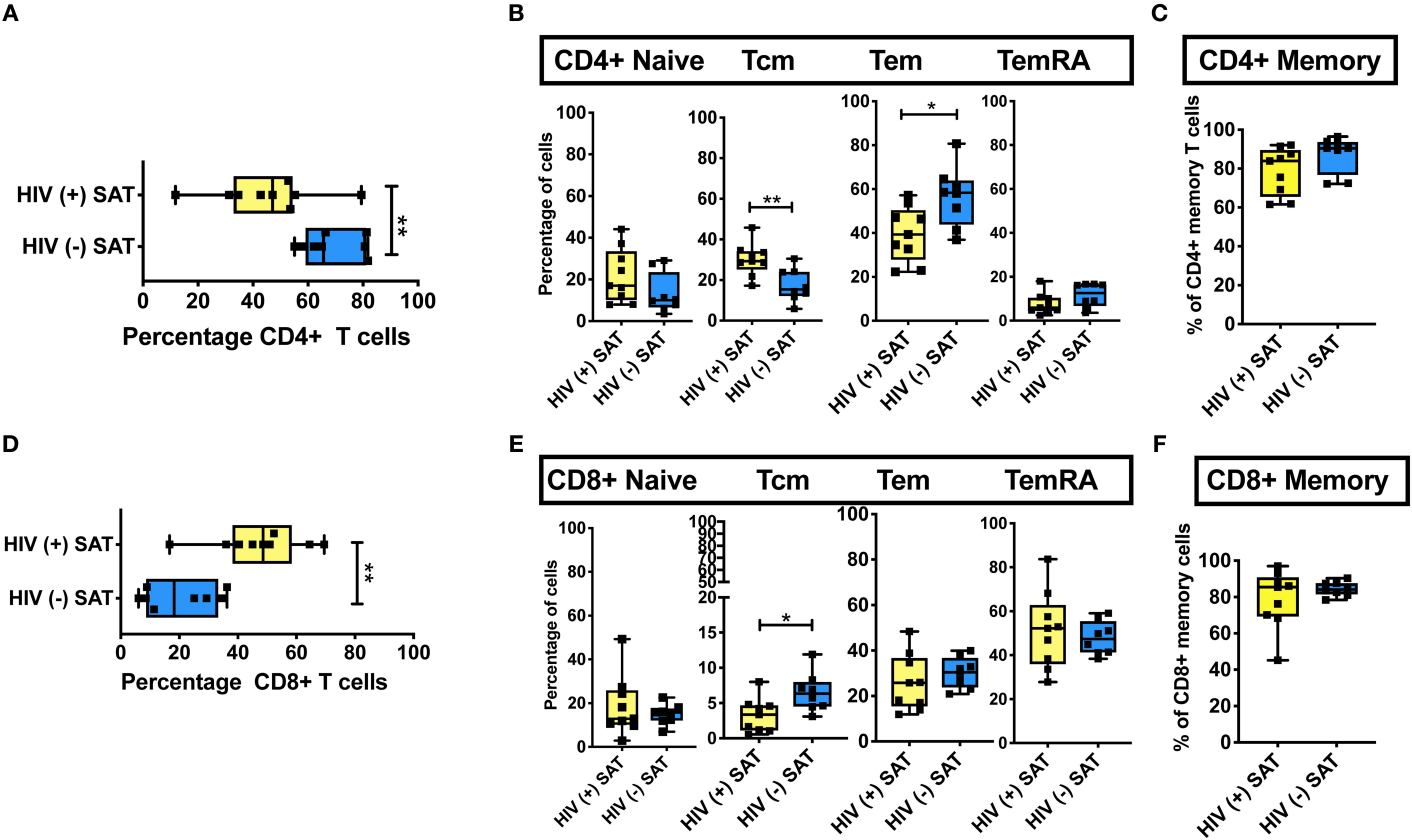
Comparison of CD4^+^ and CD8^+^ T cell subsets in subcutaneous adipose tissue of PLWH and matched HIV-negative controls. Cells recovered from subcutaneous adipose tissue (SAT) of PLWH (*n* = 9) and HIV-negative controls (*n* = 8) with similar BMI and hemoglobin A1c were compared. **(A)** CD4^+^ T cells as a percentage of total live CD3^+^ T cells. **(B)** Frequencies of T_Nai_, T_EM_, T_CM_, and T_EMRA_ cells in SAT of PLWH and HIV-negative controls. **(C)** Total CD4^+^ memory T cells (T_CM_ + T_EM_ + T_EMRA_). **(D)** Similar analysis also done on CD8^+^ T cells, which are displayed as percentage of total live CD3^+^ T cells. **(E)** Frequencies of CD8^+^ T_Nai_, T_EM_, T_CM_, and T_EMRA_ cells. **(F)** Total CD8^+^ memory T cells. The box and whiskers plot indicate mean ± SD. Mann-Whitney test used to analyze differences between unpaired PLWH and (–) subjects; ^**^p < 0.01, ^*^p < 0.05.

Phenotypic analysis of SAT memory T cell subsets in PLWH could provide insight on possible mechanisms contributing to the profound shift in the CD4:CD8 ratio that accompanies HIV infection. Therefore, we compared the proportion of CD4^+^ and CD8^+^ naive, T_CM_, T_EM_, and T_EMRA_ cells between 8 non- and pre-diabetic HIV-negative persons and 9 PLWH; these subjects were selected from the 26 PLWH in our cohort based on similar age, HbA1c and BMI values (Table 2). As in prior studies, we found that SAT from PLWH is enriched in CD8^+^ T over CD4^+^ T cells (51 vs. 47%, respectively) compared to HIV-negative persons (21 vs. 66%; Figures 6A,D). Although PLWH have a much higher proportion of CD8^+^ T cells in the SAT, we found no significant difference in the overall proportions of SAT CD4^+^ and CD8^+^ memory cells between PLWH and HIV-negative persons (Figures 6C,F), and the distribution of CD4^+^ and CD8^+^ memory T cell subsets was remarkably similar (Figures 6B,E). However, three notable differences were present: compared to PLWH, the HIV-negative persons had a significantly higher percentage of CD4^+^ T_EM_ (58 vs. 39%, *p* = 0.02), a lower percentage of CD4^+^ T_CM_ (15 vs. 29%, *p* = 0.07) in their SAT (Figure 6B), and a significantly higher percentage of CD8^+^ T_CM_ compared to PLWH (6.4 vs. 3.4%, *p* = 0.03) (Figure 6E).

These findings suggest the profound change in the SAT CD4:CD8 T cell ratio observed in PLWH is not driven by the enrichment or depletion of a single memory T cell phenotype. Rather, it is phenotype agnostic and involves shifts in disparate naïve and memory T cell phenotypes from both the CD4^+^ and CD8^+^ lineages. This raises the possibility that a chemotactic signal from SAT in PLWH is recruiting CD8^+^ T cells more robustly in the context of infection with HIV. However, the similar subset distributions suggest this signal, if present, may represent the amplification of a normal physiologic pathway as opposed to an “HIV-specific” process.

### Comparison of SAT Gene Expression in PLWH and HIV-Negative People With Similar Glucose Tolerance

**Figure 7:**
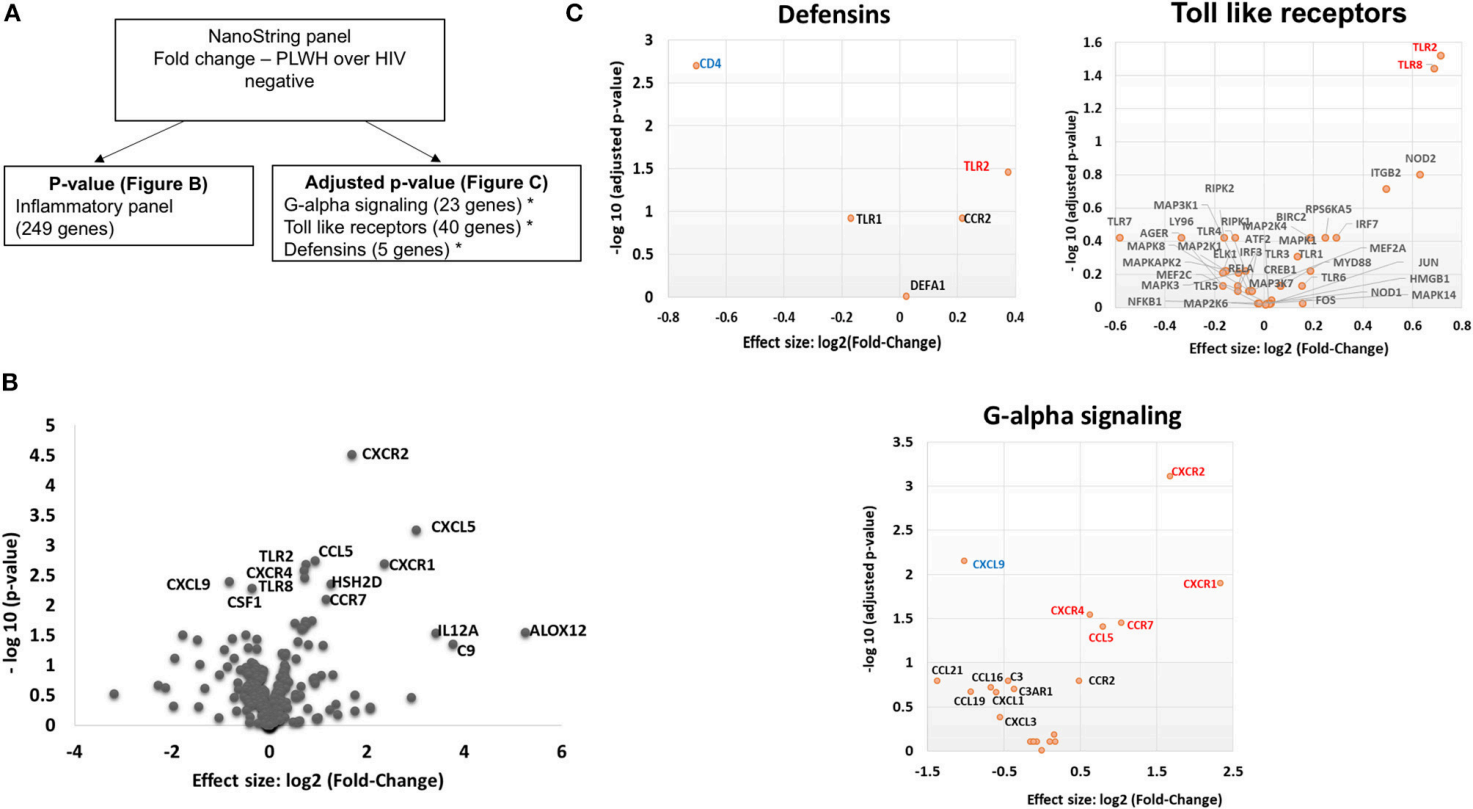
PLWH have a higher CXCR1, CXCR2, CXCR4, CCL5, CXCL5, TLR2, and TLR8 RNA expression within the subcutaneous adipose tissue compared to HIV negative individuals with similar glucose tolerance. RNA was extracted from subcutaneous adipose tissue of PLWH (*n* = 6) and HIV negative individuals (*n* = 7). **(A)** Schematic showing data analysis. **(B)** Volcano plot showing log_2_ fold change (PLWH vs. HIV negative) vs. log_10_ *p*-value. Relevant genes out of 249 genes are labeled. **(C)** Sub-groups of inflammatory gene panels available through Nanostring were compared and adjusted *p*-values calculated within these groups. Genes in G-alpha signaling, defensins and Toll-like receptors with significant findings are shown. Red denotes genes that are higher in PLWH and blue denotes higher gene expression in HIV negative.

Given the increased proportion of CD8^+^ T cells in SAT from PLWH, we performed a sub-study to investigate potentially relevant adipose tissue immune signaling pathways upregulated in the context of HIV infection. Extracted SAT mRNA from 6 PLWH and 7 HIV-negative individuals was assayed using a NanoString human inflammation panel to quantitate expression of over 250 genes representing a broad range of immune pathways (Figure 7A). The overall fold change in genes expressed from SAT of PLWH over HIV-negative is shown in the volcano plot (Figure 7B). In general, we found increased expression of chemokine receptors (CXCR2, CXCR1, CXCR4) and ligands (CCL5, CXCL5) in those with HIV. Additional genes including ALOX12 and IL12A were also elevated. We further analyzed inflammatory gene pathways defined by NanoString (Figure 7C). CXCR2, CXCR1, CXCR4, TLR2, and TLR8 gene expression were significantly higher in PLWH compared to HIV-negative (Table 4) whereas CD4 and CXCL9 were higher in HIV-negative individuals. Future studies are needed to identify the cells expressing these genes, and whether receptor and ligand expression might account for differences in the cells that traffic to SAT in PLWH.

**Table 4:** RNA gene expression in SAT of PLWH compared to HIV-negative.

**Fold change PLWH over HIV negative**						
	**Base mean**	**log2Fold** **change**	**lfcSE**	**stat**	***P*****-value**	***P*****-adj**
**GENES IN G ALPHA (I) SIGNALING EVENTS**						
CXCR2	448.38936	1.66765	0.40295	4.13858	0.00003	0.00077
CXCL9	318.40348	−1.02425	0.29993	−3.41498	0.00064	0.00702
CXCR1	64.4378	2.32789	0.74234	3.13589	0.00171	0.01256
CXCR4	614.54675	0.62417	0.22333	2.79483	0.00519	0.02856
CCR7	52.79557	1.03026	0.38811	2.65456	0.00794	0.03494
CCL5	693.45461	0.79026	0.30909	2.55671	0.01057	0.03875
**GENES IN DEFENSINS**						
CD4	429.29116	−0.70372	0.19864	−3.54278	0.0004	0.00198
TLR2	285.1579	0.37392	0.15213	2.45786	0.01398	0.03494
**GENES IN TOLL LIKE RECEPTORS**						
TLR2	301.23122	0.71396	0.21186	3.37002	0.00075	0.03007
TLR8	199.60192	0.6878	0.22039	3.12083	0.0018	0.03607
**GENES IN DISEASE**						
TLR2	302.33202	0.7476	0.20994	3.561	0.00037	0.01034
CXCR4	618.19156	0.72581	0.21895	3.31492	0.00092	0.01283

## Discussion

Several recent studies have uncovered a profound change in the balance of adipose tissue CD8^+^ and CD4^+^ T cells in PLWH, and similar studies in SIV-infected macaques indicate that the relative enrichment of CD8^+^ T cells stems from viral infection rather than from ART treatment or CD4^+^ cell depletion. However, the characteristics and consequences of this CD8^+^ cell enrichment are generally unknown. Here, we demonstrate that the adipose tissue of PLWH with viral suppression on long-term ART is a reservoir of CD4^+^ and CD8^+^ T_EM_ and T_EMRA_ cells; two cell types with high pro-inflammatory potential when stimulated. Furthermore, we show that expression of CD69, a putative marker of TCR-linked activation and tissue residency, increased on CD4^+^ T_EM_ and T_EMRA_ cells in a stepwise manner from non-diabetic, to pre-diabetic, to diabetic individuals. We also identify a phenotypically unique population of CD4^+^ T cells co-expressing CX_3_CR1, GPR56, and CD57 that is specifically enriched in the SAT of diabetic PLWH. While the significance of these cells is currently unclear, CX_3_CR1 and GPR56 have previously been identified on cells with antiviral functions (55–59). Finally, our results demonstrate that the enrichment of CD8^+^ T cells over CD4^+^ T cells in PLWH as compared to HIV-negative individuals is relatively non-specific in the sense that the change is not attributable to a profound increase or reduction in a single memory cell subset.

Our findings contribute to the growing body of literature on both adipose tissue T cells in general, as well as the immune phenotype of adipose tissue in the context of metabolic disease among PLWH. Han et al. recently demonstrated that white adipose tissue in mice is a major reservoir for memory T cells with potent proliferative, effector, and protective potential (39). The adipose tissue T cells predominantly expressed CD44, a marker of antigen experience, and were CD62L negative, which is consistent with T_EM_ and T_RM_ populations in mice. Furthermore, approximately half the CD44^+^CD62L^−^ CD8^+^ and CD4^+^ cells expressed CD69. The study also assessed recall responses of adipose tissue T cells in mice previously challenged with antigen, demonstrating an influx of monocytes and neutrophils, as well as highly reactive memory T cells. Specifically, these memory T cells indicated rapid and enhanced effector potential with upregulation of several genes involved in antimicrobial defenses. Furthermore, antigen re-challenge led to downregulation of several metabolic pathways (including lipid biosynthesis and cholesterol and long-chain fatty-acyl-CoA metabolic processes) as well as to a detectable reduction in serum levels of adiponectin and cholesterol, further highlighting the potential role memory T cells play in metabolic disease.

These animal studies suggest the accumulation of T_EM_ and T_EMRA_ cells in the adipose tissue of PLWH may represent a potent source of inflammation in the setting of antigen stimulation. Viral pathogens, including HIV and CMV, are found in adipose tissue and could serve as a chronic stimulus for T_EM_ and T_EMRA_ cells, with downstream effects on metabolic function (17, 21, 39, 51). Our observation of increasing T_EM_ and T_EMRA_ CD69 expression with declining glucose tolerance in PLWH may indicate the enrichment of a resident memory phenotype in diabetic individuals. Our study design precludes an assessment of whether the presence of increased CD69^+^ T_EM_ and T_EMRA_ cells preceded or followed the development of glucose intolerance, though future longitudinal studies in PLWH with early indications of metabolic disease could address this question. Furthermore, future studies to identify the receptor specificity of the CX_3_CR1, GPR56, and CD57-expressing CD4^+^ cells, and experiments to co-culture these cells with adipocytes, may help characterize the role of these immune cells in adipose tissue, identify their cytokine expression patterns, and explore the potential effects of these cells on adipocyte function.

An early study of SAT and VAT from PLWH by Couturier et al. identified major differences in CD4^+^ and CD8^+^ T cell populations compared to HIV-negative controls (17), which were subsequently reported in other HIV and SIV studies (18, 19, 21). In the HIV-negative controls, adipose tissue SVF CD3^+^ T cells in SAT were predominantly memory CD4^+^ CD45RO^+^ T cells (61%) with fewer memory CD8^+^ T cells (15%), while this distribution was reversed in PLWH, with more memory CD8^+^ T cells (46%) compared to memory CD4^+^ T cells (35%). This represented an ~50% enrichment in memory CD8^+^ T cells over the blood in the subjects with HIV and was not reflective of differences in peripheral blood T cell subsets between the two groups.

Notably, the Couturier et al. study found significant differences in the rates of CD69 expression on memory CD4^+^ and CD8^+^ T cells in adipose tissue vs. blood: <5% of these cells expressed CD69 in the blood, compared to 60–67% in adipose from PLWH and 61–72% in the adipose from HIV negative persons. A similar study comparing activation markers on adipose tissue T cells in HIV-negative lean, overweight and obese individuals using fine needle aspiration found ~5–10% CD69 expression on SAT CD4^+^ T cells and ~25% expression on CD8^+^ T cells (60). We observed the highest CD69 expression on T_EM_ cells in diabetic PLWH (mean 22%). This was significantly higher than CD69 expression on TEM cells from non-diabetics (mean 15%) and pre-diabetics (mean 18%), and 20-fold higher compared to TEM cells in blood. The reason for the lower CD69 expression in our cohort compared to Couturier et al. is unclear and may reflect differences in CD69 expression in adipose tissue samples collected after death or by surgical resection as opposed to liposuction aspirates processed within 30–60 min of collection in our study, differences in tissue processing to extract T cells, or could be explained by residual peripheral blood in samples obtained via liposuction.

Prior studies have also demonstrated a significantly higher proportion of SAT Treg cells (defined as CD25^+^ FOXP3^+^ CD4^+^ T cells; a cell type thought to exert an anti-inflammatory effect and reported to be depleted in obese adipose tissue) in PLWH compared to HIV-negative persons, with no major differences in T_H_1 and T_H_17 pro-inflammatory subsets (19, 24). The proportion of T_H_1 CD4^+^ T cells (expressing intermediate or high levels of T-bet) did not differ according to HIV status, while T_H_2 CD4^+^ T cells (expressing GATA-3) were barely detected in the adipose tissue from both PLWH and HIV-negative persons. While CD4^+^ T cell expression of HLA-DR (24%) in SAT was higher compared to blood in the HIV-negative, similar levels of SAT HLA-DR expression were observed in the PLWH. Furthermore, SAT CD4^+^ T cell expression of PD-1 expression was much higher compared to PBMCs (45 vs. 3%), but again there were no significant differences in SAT according to HIV status (19).

Two studies of SIV-infected cynomolgus macaques confirm the adipose tissue CD8^+^ T cell enrichment observed in PLWH is a viral phenomenon, rather than related to ART treatment (18, 61). In both studies, SIV infection was associated with a higher percentage of CD8^+^ T cells in both the SAT and VAT compared to non-infected animals (18, 61). One study also demonstrated the inverted CD8:CD4 ratio was not driven by a reduction in the total number of CD4^+^ T cells in infected animals; rather, SIV-infected animals had significantly higher density of CD8^+^ T cells in VAT and a somewhat higher density in SAT (18). For both non-infected healthy and SIV-infected monkeys, the majority of the adipose tissue CD4^+^ and CD8^+^ T cells were memory T cells (>94% CD95^+^), with a large fraction of activated cells marked by expression of CD69^+^ (62–84%) and CD25^+^ (3–13%).

A central question that remains unanswered is whether the increase in the proportion of adipose tissue CD8^+^ T cells in PLWH and macaques with SIV results from *in situ* proliferation vs. increased infiltration from the circulation. The high expression of CD69 on SAT memory cells suggests a “tissue resident” phenotype, but does not answer the question of whether these cells are expanded clones or prior migrants. On the one hand, a study by Damouche et al. found no significant differences in the proportion of CD4^+^ or CD8^+^ T cells expressing Ki-67, indicative of cycling and recently divided cells, between PLWH vs. HIV-negative controls (19). The authors suggested the low percentage of Ki-67^+^ cells in SAT (<2%) from PLWH and HIV-negative subjects reflected minimal T cell proliferation within the tissue. Similarly, a study in macaques found no differences in proportions of Ki-67-expressing CD4^+^ or CD8^+^ T cells in animals with and without SIV, suggesting the higher density of CD8^+^ T cells in adipose tissue does not result from recent proliferation (18). However, proliferation of memory T cells within adipose tissue is clearly demonstrated in other studies. Han et al. injected mice with pseudotuberculosis and evaluated memory T cells 4 weeks later. They showed proliferation of T_EM_ and T_RM_ by Ki67 expression, bromodeoxyuridine labeling, and cell cycle stage analysis of memory T cells (39).

The use of T cell receptor (TCR) sequencing of adipose tissue T cell subsets in future studies may provide insight into the clonal lineage of adipose tissue T cells. Previously, our group demonstrated that CD8^+^ TCR clonality is higher in SAT compared to blood in PLWH using bulk TCRβ CDR3 deep sequencing, where bias-controlled V and J gene primers are used to amplify rearranged V(D)J segments (21, 62, 63). In that study, the 10 most prevalent TCRβ clones comprised a significantly larger percentage of total clones in SAT (25%) compared to paired blood (16%), and the Shannon's Entropy index, a measure of overall repertoire diversity, was lower in adipose tissue compared to blood (4.39 vs. 4.46, respectively). Additionally, V-J gene pairing and gene usage differed between blood and adipose tissue, albeit not statistically significant, potentially due to the small sample size. While these findings are intriguing, the lower proportion of CD4^+^ and CD8^+^ T_Nai_ cells in SAT demonstrated in the current analysis may have had a role in the higher SAT clonality scores reported in the prior study. In addition to clonal lineage, TCR sequencing may also inform our understanding of potential antigen targets for adipose tissue T cells in PLWH. A recent study in mice demonstrated that diet-induced obesity is characterized by increased adipose tissue CD8^+^ T cell density, and the TCR repertoire of these CD8^+^ T cells is more clonal and positively charged (in respect to amino acids). This work was also the first to demonstrate that isolevuglandins (a group of negatively charged reactive gamma-ketoaldehydes generated by free radical oxidation) presented on adipose tissue macrophages from obese fat can independently activate T cells, potentially highlighting a mechanism contributing to inflammation (25). Examining TCR charge and polarity, in addition to clonality, and investigating antigen presenting cells in the adipose tissue may be an important approach for further characterizing the adipose tissue immune milieu in PLWH vs. HIV-negative persons.

Profiling of adipose tissue mRNA expression in SIV-infected macaques showed significantly higher levels of IL-2, IL-7, and CCL19 in acute SIV compared to uninfected controls, which may contribute to the homing and survival of T cells (61). In our gene expression sub-study on whole adipose tissue of PLWH and HIV-negative controls with similar glucose tolerance, we found >2-fold expression of IL12A, CXCR1, CXCL5, ALOX12, and C9 in the PLWH compared to HIV-negative. Analysis of subgroups of inflammatory gene pathways revealed significantly higher levels of TLR2, TLR8, CXCR4, CCR7, CCL5, CXCR1, and CXCR2 and lower levels of CXCL9 and CD4 in the PLWH.

Previous studies have linked inducible and increased expression of TLR2 in PBMC and SAT of obese individuals with type 2 diabetes (64–67). TLR8 is an endosomal receptor that binds HIV-1 single-stranded RNA and is similar to TLR2 in its MyD88-dependent activation of NF-kB. Previous studies of TLR8 expression indicate that it is most often absent or present at low levels in adipose tissue (64), but that a positive correlation exists between plasma CRP levels and adipose tissue TLR8 expression. In our study, we are unable to discriminate whether the differences in TLR expression are due to alterations on T cells, macrophages, adipocytes or pre-adipocytes. However, we likely have identified a contribution of TLR8 to SAT inflammation that is more pronounced in PLWH compared to HIV-negative individuals with similar glucose tolerance and BMI.

Regarding chemokine ligands and receptors, M1 macrophages express higher levels of CCR7, CCL9, and CCL5 and lower levels of CXCR4 in comparison to M2 macrophages (68). In the context of HIV infection, CXCR1 and CXCR2 are of interest because HIV-1 matrix protein p17 has been shown to mimic IL-8 and binds CXCR2 with high affinity, stimulating pro-angiogenic ERK downstream (69). Angiogenesis has been linked to obesity via neovascularization-driven migration of adipocytes (70). Further studies of chemokine receptors on CD4^+^ and CD8^+^ T cells may provide insight into whether HIV infection results in signals that preferentially recruit CD8^+^ T cells over CD4^+^ T cells independent of obesity.

## Limitations

Our cohort study was cross-sectional and enrolled a total of 34 participants. We classified participants as non-diabetic and pre-diabetic based on fasting blood glucose and HbA1c values as recommended for clinical practice by the American Diabetes Association (diabetics were classified based on medication usage). In the future, the measurement of fasting insulin and use of the homeostatic model assessment (HOMA) may provide more nuanced stratification of participants. While a strength of our study was the collection of SAT via liposuction and rapid processing to extract T cells, samples may have contained residual peripheral blood, which would bias our comparisons of SAT and blood toward the null hypothesis. We were limited in the number of markers we could examine via flow cytometry and did not examine additional markers of tissue resident cells, as well as T regulatory cells and T_H_1/T_H_2 subsets. Our gene expression analysis of SAT from PLWH and HIV-negative persons was on whole adipose tissue as opposed to sorted SVF cells. Therefore, differences in genes cannot be directly linked to specific cell types.

## Conclusions

Although PLWH can survive decades on effective ART, this success is offset by the rising burden of metabolic diseases affecting the HIV population (1–3). Here, we demonstrate that the adipose tissue of PLWH is a reservoir of CD4^+^ and CD8^+^ T_EM_ and T_EMRA_ cells; two cell types with high pro-inflammatory potential when stimulated. Furthermore, we show that expression of CD69, a putative marker of TCR-linked activation and tissue residence, on CD4^+^ T cells increases in a stepwise manner from non-diabetic, to pre-diabetic, to diabetic individuals, which may reflect an interaction between cells of the adaptive immune system and adipocytes in the context of metabolic disease. We also identify a population of CD57^+^CX_3_CR1^+^GPR56^+^ co-expressing CD4^+^ T cells that is specifically enriched in the SAT of diabetics. This could represent a group of virus-specific cells that contribute to inflammation in adipose tissue and potentially pre-dispose PLWH to metabolic disease, though further studies to assess the effects of these cells on adipocytes are needed. Finally, our results demonstrate that the enrichment of CD8^+^ T cells over CD4^+^ T cells in PLWH as compared to HIV-negative individuals is relatively non-specific in the sense that the change is not attributable to a profound increase or reduction in a single memory cell subset. Further studies are needed to understand the clonal lineage, TCR characteristics, antigen targets, chemokine receptors and the functional phenotype of CD57^+^CX_3_CR1^+^GPR56^+^ T cells in the adipose tissue of PLWH, and to identify potential therapeutic targets.

## Data Availability

The datasets generated for this study are available on request to the corresponding author.

## Ethics Statement

The study was carried out in accordance with the recommendations of the human experimentation ethical standards of the Vanderbilt Institutional Review Board. All subjects gave written informed consent, in accordance with the Helsinki Declaration of 1975, as revised in 2000. The protocol was approved by the Vanderbilt Institutional Review Board.

## Author Contributions

CW, WM, and JK: conceptualization and methodology; WM and RR: software; CW, WM, MP, and JK: validation; WM, CW, RF, and YF: formal analysis; CW, WM, LB, JS, BW, BF, ML, MM, PB, and RR: investigation; JK, SK, and SM: resources; WM and CW: data curation; CW, WM, and JK: writing—original draft; CW, WM, and RR: visualization; JK, SM, and SK: supervision; CW, WM, JK, RF, and YF: statistics; JK, NB, MP, and SK: project administration; JK and SM: funding acquisition; All authors: writing—review and editing.

### Conflict of Interest Statement

The authors declare that the research was conducted in the absence of any commercial or financial relationships that could be construed as a potential conflict of interest.
