# Supplementary material for: Adipose Tissue in Persons With HIV Is Enriched for CD4+ T Effector Memory and T Effector Memory RA+ Cells, Which Show Higher CD69 Expression and CD57, CX3CR1, GPR56 Co-expression With Increasing Glucose Intolerance
Source: Front Immunol. 2019 Mar 19;10:408. doi: 10.3389/fimmu.2019.00408 (PMC6433850; doi:10.3389/fimmu.2019.00408)
Supplement: Supplementary file 1 [file Data_Sheet_1.PDF]

Supplementary Figure legend

Supplementary Figure 1. **Study groups and flow cytometry gating panels**. (A) Study subjects were placed in four groups. Matched cryopreserved PBMC and SAT were analyzed from subjects from Group 1 (n=9), Group 2 (n=8), and Group 3 (n=9). (B) Representative plots showing multi-parametric flow cytometry gating panels used to analyze $CD3^+$, $CD4^+$ and $CD8^+$ T cells in matched PBMC and SAT. (C) Memory CD4 and CD8 T cells were assessed using CCR7 and CD45RO surface markers (TEM: $CCR7^-CD45RO^+$; TCM: $CCR7^+CD45RO^+$; TEMRA: $CCR7^-CD45RO^-$).

Supplementary Figure 2. Adipose Tissue has a higher percentage of TEM ($CD45RO^+$ $CCR7^-$) and TEMRA ($CD45RO^-$ $CCR7^-$) cells compared to peripheral blood. The average of all four subsets for 26 subjects are represented as number of cells (each colored circle) per 100 T cells in each group.

Supplementary Figure 3. Unsupervised analysis showing distribution of $CD4^+$ and $CD8^+$ subsets compared to matched PBMCs. Representative plots showing analysis performed on gated $CD3^+$ T cells in PBMCs and SAT cells. viSNE analysis illustrates the distribution of memory (TCM, TEM, TEMRA) and naïve $CD8^+$ and $CD4^+$ T cell subsets within gates $CD3^+$ T cells.

Supplementary Figure 4. Analysis of $CD4^+$ and $CD8^+$ memory subsets and BMI. A) The graphs on the top row show frequencies of T_{Nai}, T_{EM}, T_{CM} and T_{EMRA} $CD4^+$ cells in SAT and PBMC from all twenty-six subjects. Plots separated based on BMI (<30 [n=4], 30-35 [n=12], > 35 kg/m^2 [n=10]. B) The bottom row shows frequencies of $CD8^+$ T_{Nai}, T_{EM}, T_{CM} and T_{EMRA} cells in adipose tissue and PBMC according to BMI. The box and whiskers plot indicate mean $\underline{+}$ SD. Wilcoxon matched pair signed test was used to calculate statistics between matched PBMC and adipose pairs; Mann-Whitney test was used to analyze differences in SAT and PBMC T cell subsets between metabolic groups; ***< 0.001, **< 0.01, * p < 0.05.

Supplementary Figure 5. $CD4^+$ and $CD8^+$ TEM proportions in SAT increase with age while naïve T cells decrease with age. A) Frequencies of $CD4^+$ T_{Nai}, T_{EM}, T_{CM} and T_{EMRA} cells in adipose tissue and PBMC from all twenty-six subjects. Plots separated based on Age (<35 [n=4], 35-55 [n=12], > 56 years [n=11]). B) The bottom row shows frequencies of $CD8^+$ T_{Nai}, T_{EM}, T_{CM} and T_{EMRA} cells in SAT and PBMC. The box and whiskers plot indicate mean $\underline{+}$ SD. Wilcoxon matched pair signed test was used to calculate statistics between matched PBMC and adipose pairs; Mann-Whitney test used to analyze differences in SAT and PBMC T cell subsets between metabolic groups; ** p< 0.01, * p < 0.05.

Supplementary Figure 6. $CD4^+$ T cells co-expressing CD57, CX_3CR1, GPR56 in PBMC are higher in pre-diabetic and diabetic PLWH. A) Visualization of groups of $CD4^+$ T_{Nai}, TEM, TCM and TEMRA cells from peripheral blood B) Concatenated viSNE plots of non-diabetic (n=7), pre-diabetic (n= 5) and Diabetic (n=6) PLWH showing clusters of cells expressing CX_3CR1, CD57, GPR56 and CD69 C) Violin plots showing percentage of $CD4^+$ total, TEM and $^+$TEMRA cells co-expressing CD57, CX_3CR1 and GPR56. Mann-Whitney test used to analyze differences between unpaired samples; ** P < 0.01, * P < 0.05.

Supplementary Figure 7. $CD8^+$ T cells co-expressing CD57, CX_3CR1, GPR56 in SAT of PLWH. A) Representative plot showing clusters of naïve, TEM, TCM and TEMRA $CD8^+$ T from SAT. B) Concatenated

viSNE plots of non-diabetic (n=7), pre-diabetic (n= 5) and Diabetic (n=6) PLWH showing clusters of cells expressing CX3CR1, CD57, GPR56 and CD69.

Supplementary Figure 8. CD4$^+$ and CD8$^+$ T cells co-expressing CD57, CX$_3$CR1, GPR56 in SAT of HIV-negative persons. A) Representative plot showing clusters of naïve, TEM, TCM and TEMRA CD4$^+$ T from SAT and Concatenated viSNE plots (n=8) showing clusters of cells expressing CX3CR1, CD57, GPR56 and CD69. B) Representative plot showing clusters of naïve, TEM, TCM and TEMRA CD8$^+$ T from SAT and Concatenated viSNE plots (n=8) showing clusters of cells expressing CX3CR1, CD57, GPR56 and CD69.

Supplementary Figure 9. Representative plots showing event count distribution. A) SAT from diabetic subject showing number of events within each gate and as percentage of parent or total. Two-dimensional plots below are matched with gates showing where CD4+ and CD8+ T cell subsets fall within four quadrants. B) Gating of matched PBMC.

Supplementary Figure 10. Event counts of individual SAT and PBMC samples from all twenty-six subjects. A) Ungated cells B) Lymphocytes and C) Live CD3$^+$ T cells.

Supplementary Figure 11. CD57 expression on CD4, CD8 T cells and CD69 expression on CD8+ T cells. A) Total CD8$^+$ T, T_{Nai} , T_{EM}, T_{CM} and T_{EMRA} cells expressing the CD69 marker, and B) Frequencies of total CD4$^+$ cells and subsets expressing CD57 and C) total CD8$^+$ cells and subsets expressing CD57. The box and whiskers plot indicate mean $\pm$ SD. Wilcoxon matched-pair rank test was used to calculate differences between PBMC and SAT. Mann-Whitney test used to calculate differences between groups; blue lines and red * depict differences between groups** $p < 0.01$, * $p < 0.05$.
