# Supplementary figures and images for: Adipose Tissue in Persons With HIV Is Enriched for CD4+ T Effector Memory and T Effector Memory RA+ Cells, Which Show Higher CD69 Expression and CD57, CX3CR1, GPR56 Co-expression With Increasing Glucose Intolerance
Source: Front Immunol. 2019 Mar 19;10:408. doi: 10.3389/fimmu.2019.00408 (PMC6433850; doi:10.3389/fimmu.2019.00408)

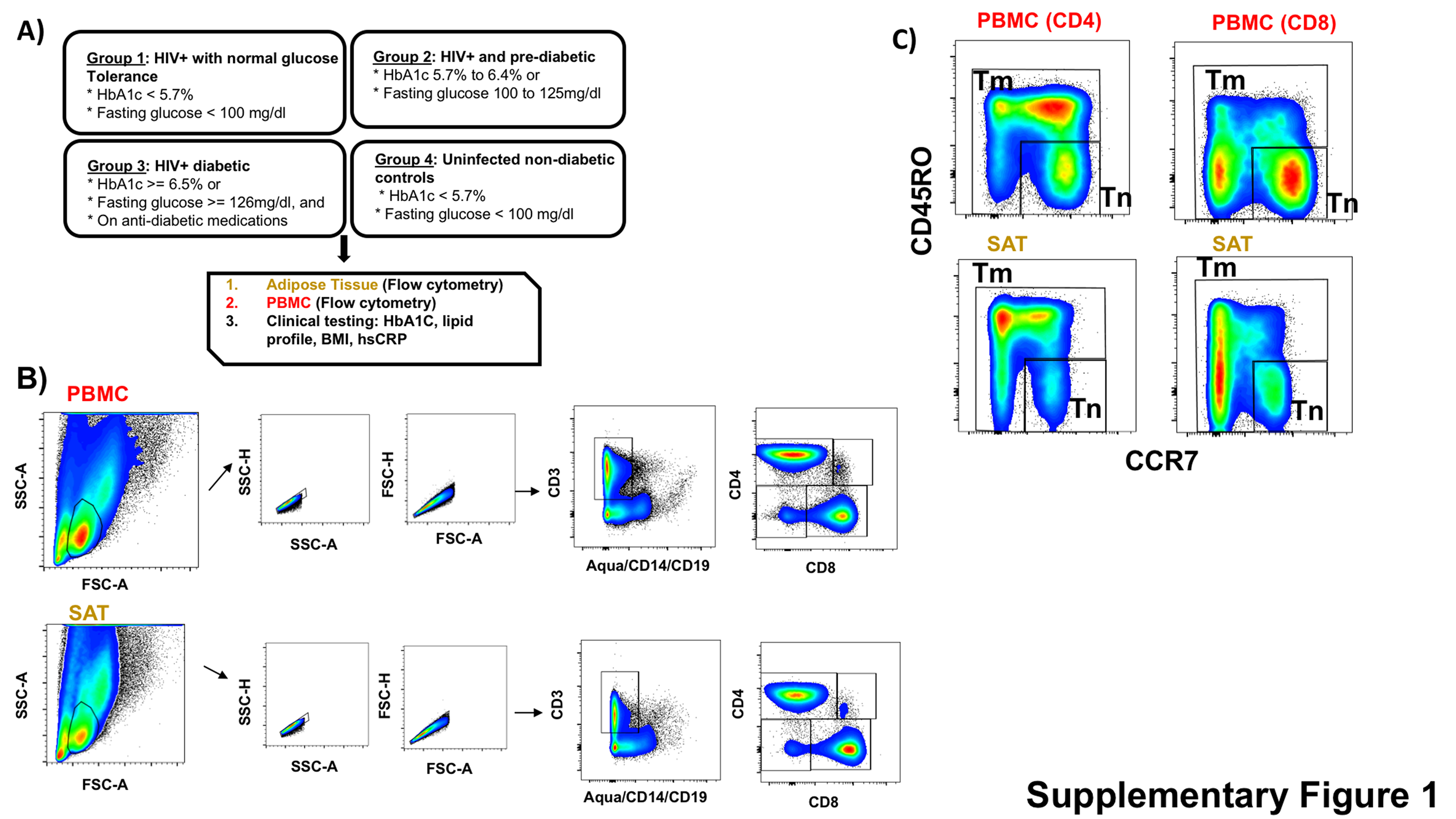

Supplement: Supplementary file 2 [file Image_1.TIFF]

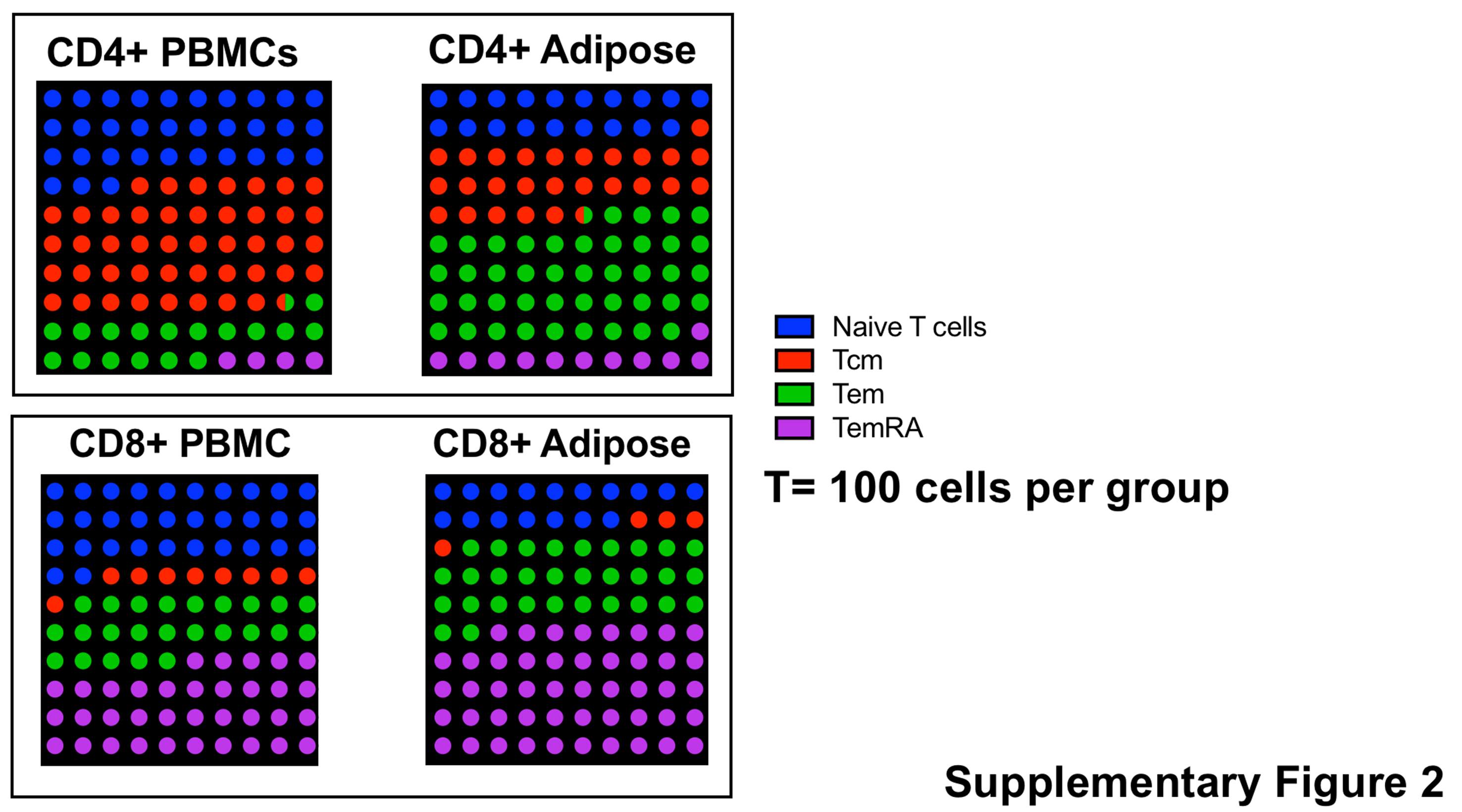

Supplement: Supplementary file 3 [file Image_2.TIFF]

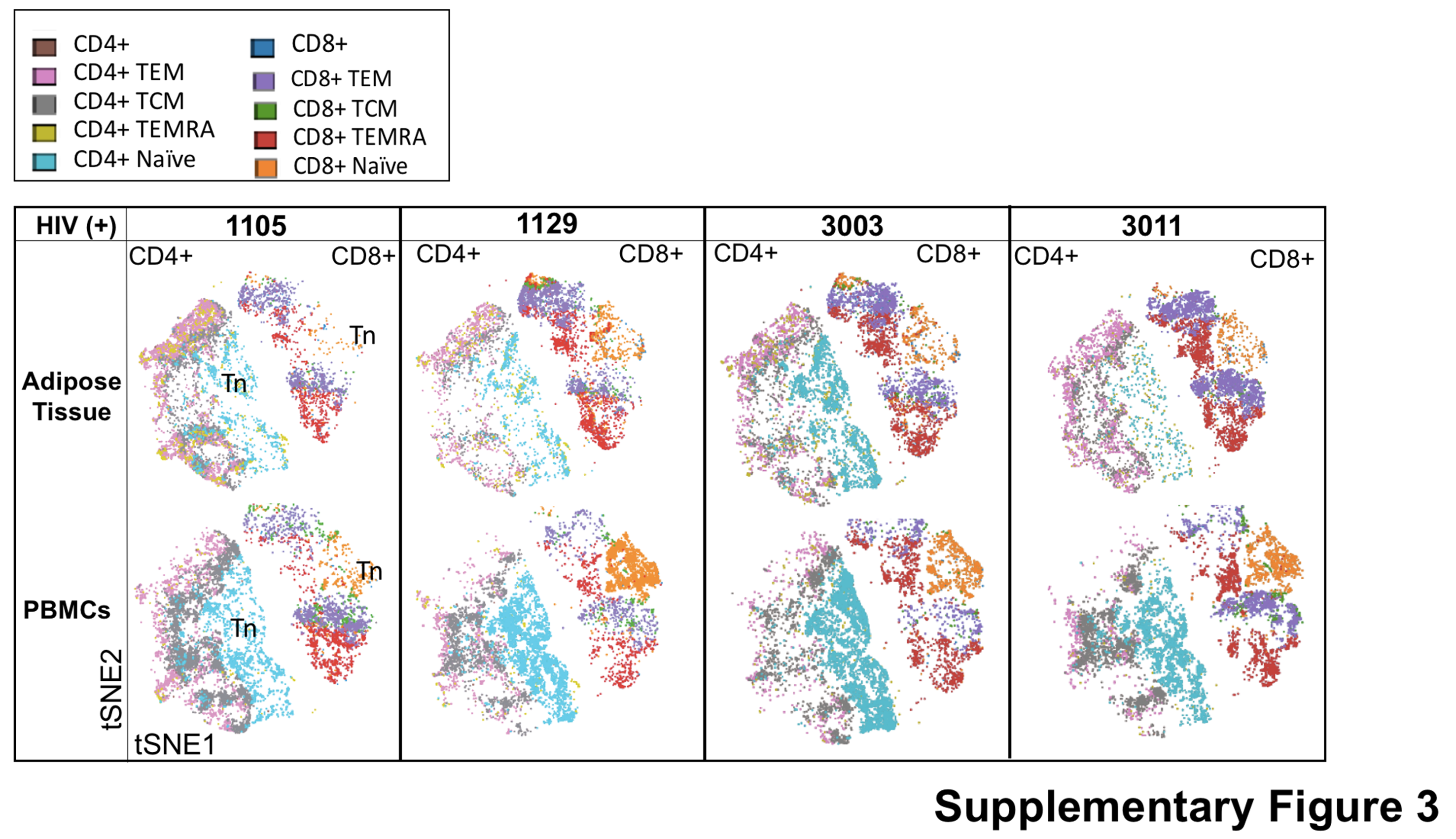

Supplement: Supplementary file 4 [file Image_3.TIFF]

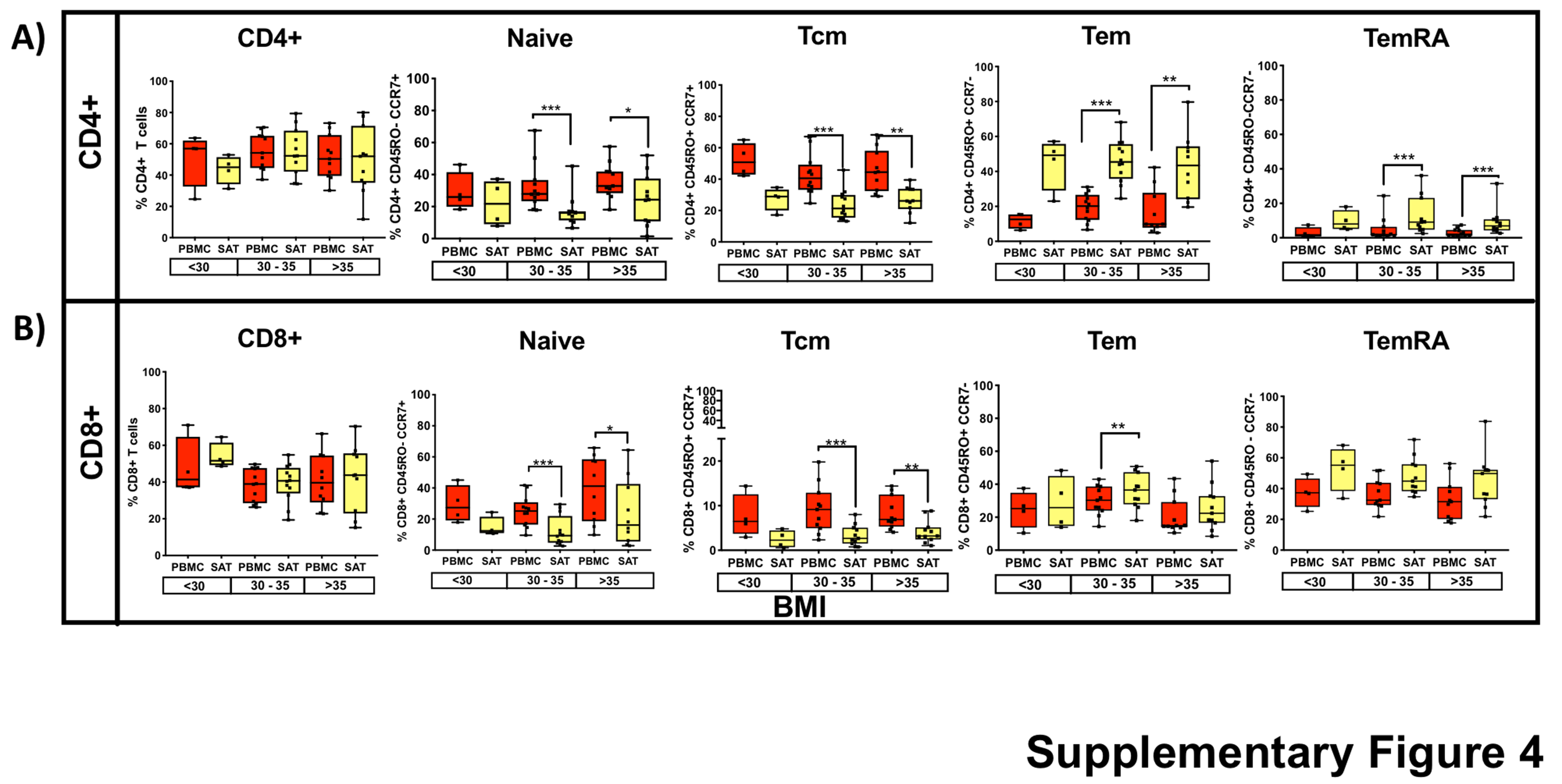

Supplement: Supplementary file 5 [file Image_4.TIFF]

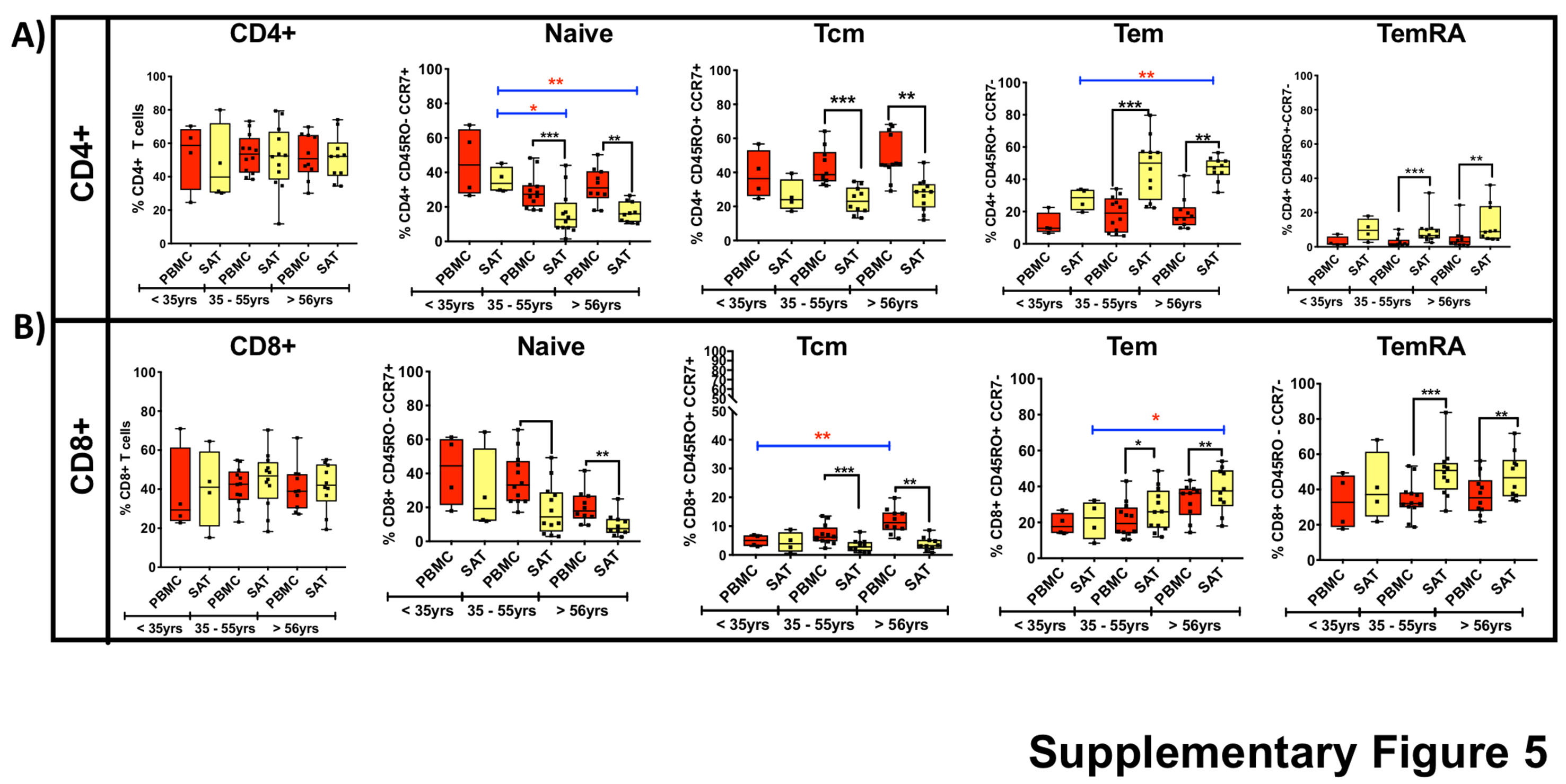

Supplement: Supplementary file 6 [file Image_5.TIFF]

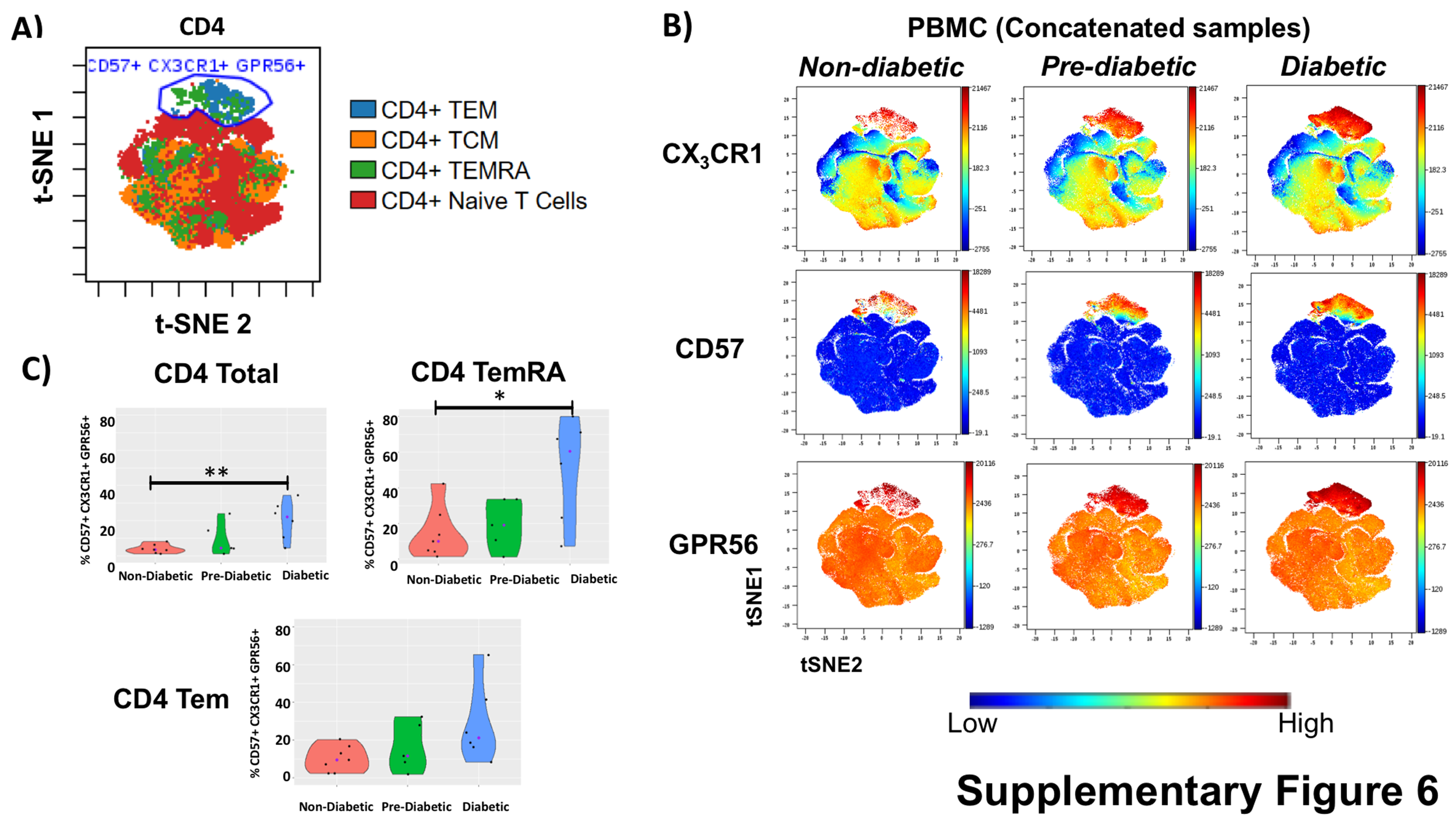

Supplement: Supplementary file 7 [file Image_6.TIFF]

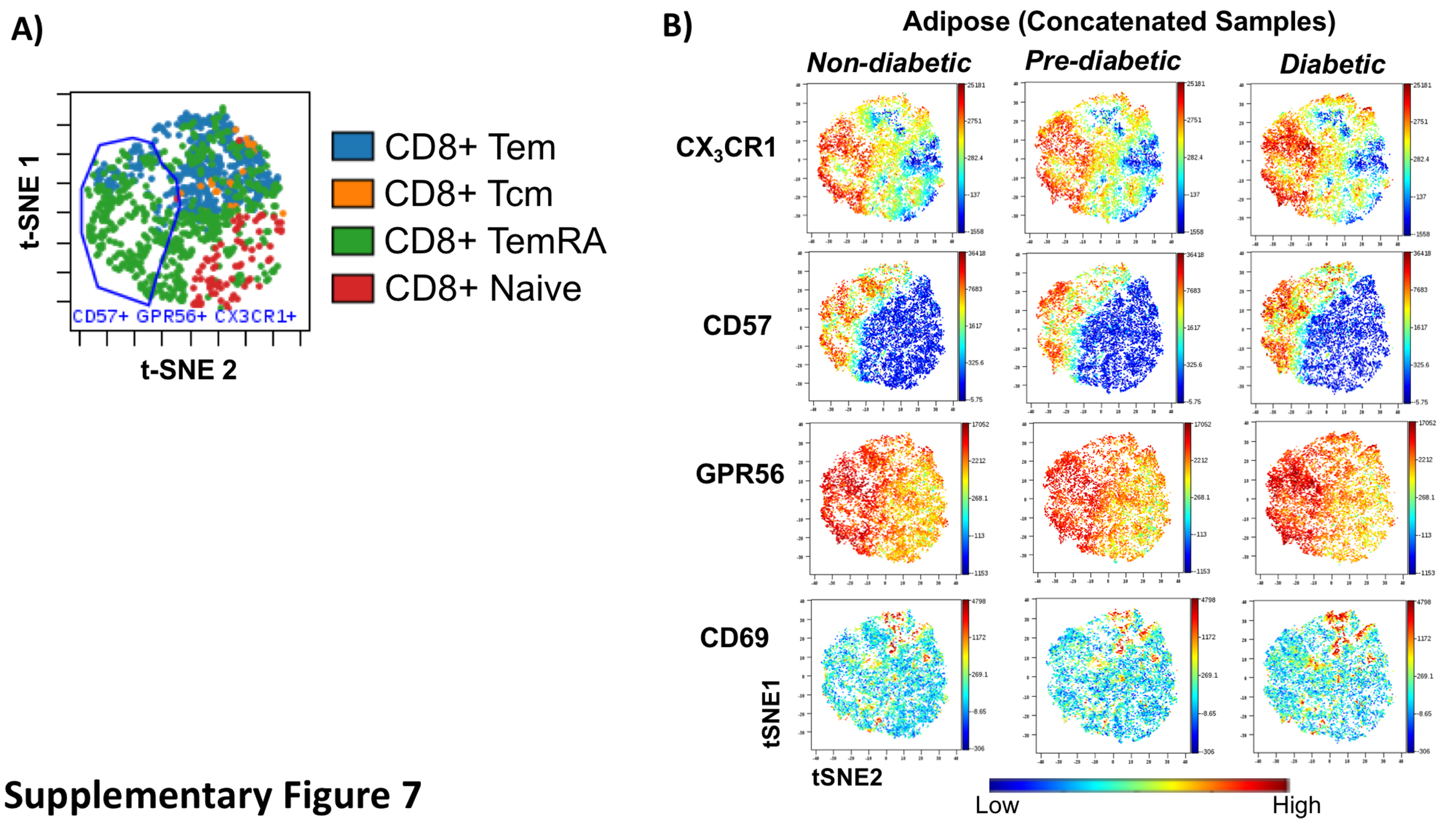

Supplement: Supplementary file 8 [file Image_7.TIFF]

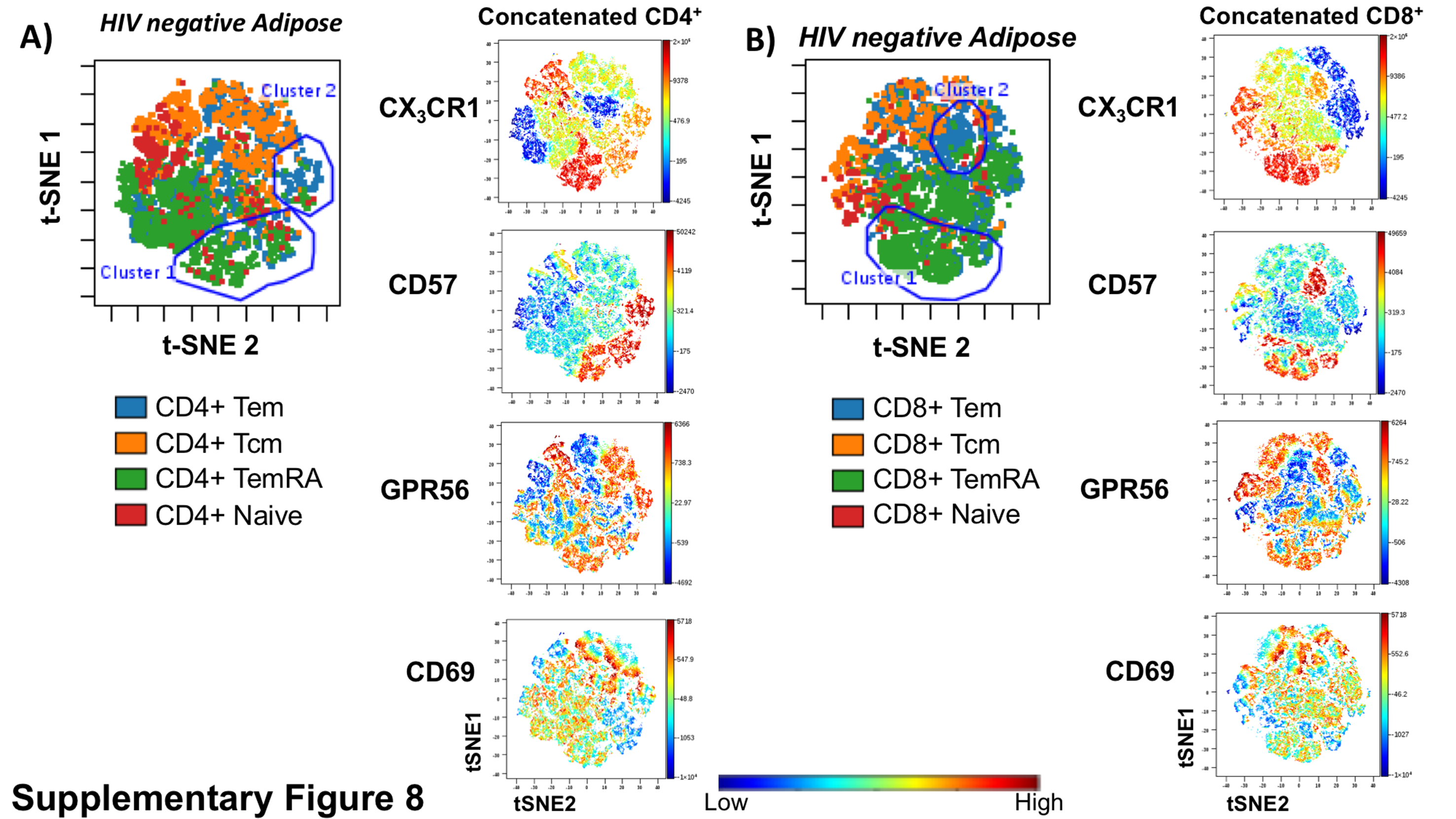

Supplement: Supplementary file 9 [file Image_8.TIFF]

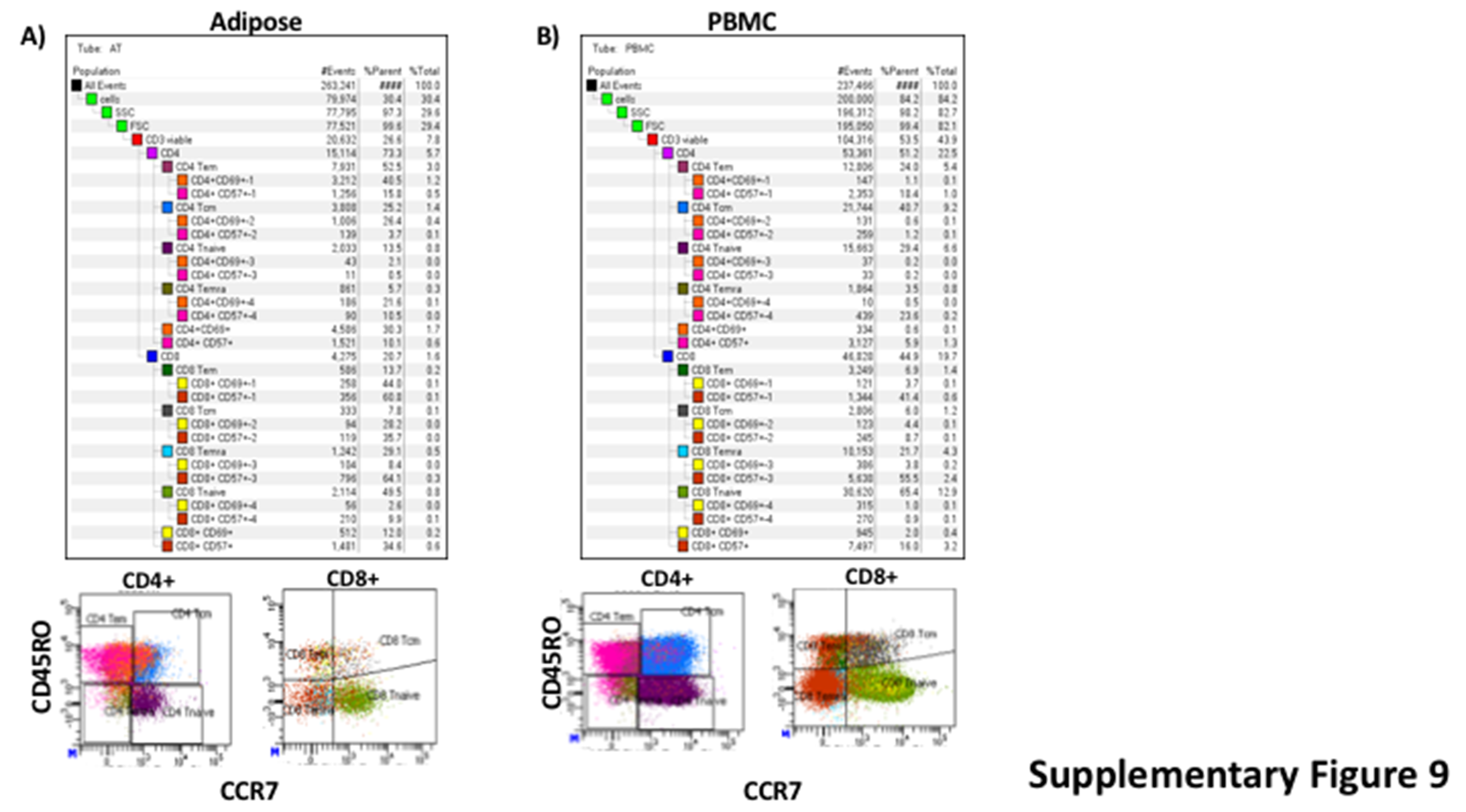

Supplement: Supplementary file 10 [file Image_9.TIFF]

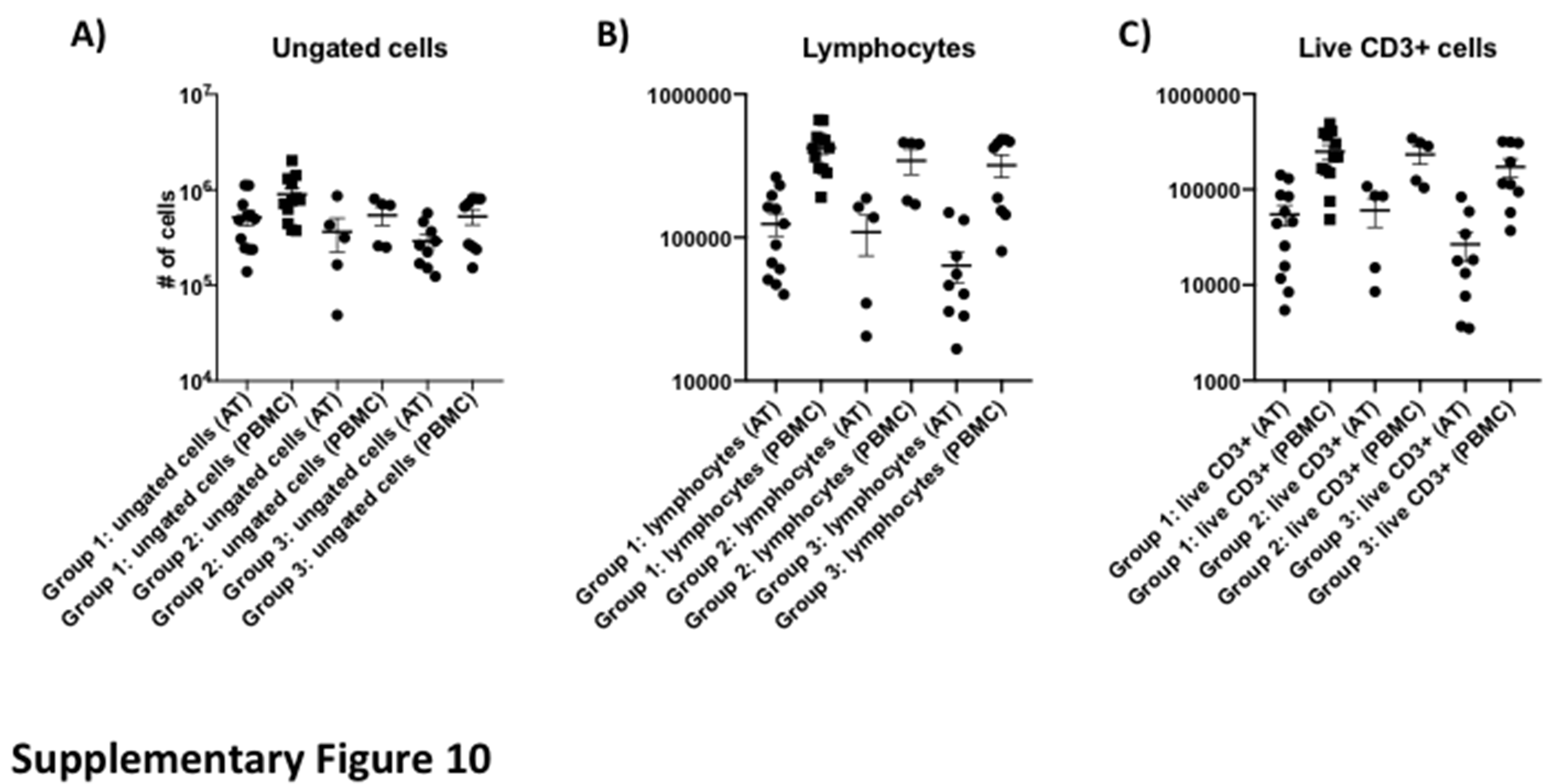

Supplement: Supplementary file 11 [file Image_10.TIFF]

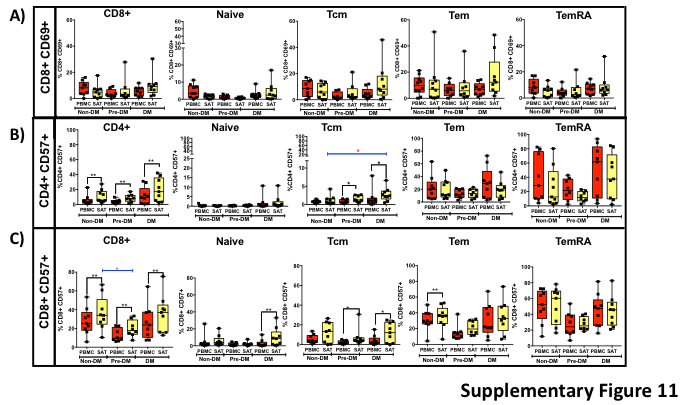

Supplement: Supplementary file 12 [file Image_11.TIFF]
